# Stroma Transcriptomic and Proteomic Profile of Prostate Cancer Metastasis Xenograft Models Reveals Prognostic Value of Stroma Signatures

**DOI:** 10.3390/cancers12123786

**Published:** 2020-12-15

**Authors:** Sofia Karkampouna, Maria R. De Filippo, Charlotte K. Y. Ng, Irena Klima, Eugenio Zoni, Martin Spahn, Frank Stein, Per Haberkant, George N. Thalmann, Marianna Kruithof-de Julio

**Affiliations:** 1 Urology Research Laboratory, Department for BioMedical Research, University of Bern, Murtenstrasse 35, 3008 Bern, Switzerland; sofia.karkampouna@dbmr.unibe.ch (S.K.); mariarosaria.defilippo@unibas.ch (M.R.D.F.); irena.klima@dbmr.unibe.ch (I.K.); eugenio.zoni@dbmr.unibe.ch (E.Z.); george.thalmann@insel.ch (G.N.T.); 2 Oncogenomics Laboratory, Department for BioMedical Research, University of Bern, Murtenstrasse 40, 3008 Bern, Switzerland; charlotte.ng@dbmr.unibe.ch; 3 Lindenhofspital Bern, Prostate Center Bern, 3012 Bern, Switzerland; martin.spahn@hin.ch; 4 Proteomics Core Facility, EMBL Heidelberg, Meyerhofstraße 1, 69117 Heidelberg, Germany; frank.stein@embl.de (F.S.); per.haberkant@embl.de (P.H.); 5 Department of Urology, Inselspital, Anna Seiler Haus, Bern University Hospital, 3010 Bern, Switzerland

**Keywords:** prostate cancer, stroma signature, patient-derived xenografts

## Abstract

**Simple Summary:**

Currently, there is a need for prognostic tools that can stratify patients, who present with primary disease, based on whether they are at low or high risk for drug resistant and hormone-independent lethal metastatic prostate cancer. The aim of our study was to assess the potentially added value of tumor microenvironment (stroma) components for the characterisation of prostate cancer. By utilising patient derived-xenograft models we show that the molecular properties of the stroma cells are highly responsive to androgen hormone levels, and considerable ECM remodelling processes take place not only in androgen-dependent but also in androgen-independent tumor models. Transcriptomic mechanisms linked to osteotropism are conserved in bone metastatic xenografts, even when implanted in a different microenvironment. A stroma-specific gene list signature was identified, which highly correlates with Gleason score, metastasis progression and progression-free survival, and thus could potentially complement current patient stratification methods.

**Abstract:**

Resistance acquisition to androgen deprivation treatment and metastasis progression are a major clinical issue associated with prostate cancer (PCa). The role of stroma during disease progression is insufficiently defined. Using transcriptomic and proteomic analyses on differentially aggressive patient-derived xenografts (PDXs), we investigated whether PCa tumors predispose their microenvironment (stroma) to a metastatic gene expression pattern. RNA sequencing was performed on the PCa PDXs BM18 (castration-sensitive) and LAPC9 (castration-resistant), representing different disease stages. Using organism-specific reference databases, the human-specific transcriptome (tumor) was identified and separated from the mouse-specific transcriptome (stroma). To identify proteomic changes in the tumor (human) versus the stroma (mouse), we performed human/mouse cell separation and subjected protein lysates to quantitative Tandem Mass Tag labeling and mass spectrometry. Tenascin C (TNC) was among the most abundant stromal genes, modulated by androgen levels in vivo and highly expressed in castration-resistant LAPC9 PDX. The tissue microarray of primary PCa samples (*n* = 210) showed that TNC is a negative prognostic marker of the clinical progression to recurrence or metastasis. Stroma markers of osteoblastic PCa bone metastases seven-up signature were induced in the stroma by the host organism in metastatic xenografts, indicating conserved mechanisms of tumor cells to induce a stromal premetastatic signature. A 50-gene list stroma signature was identified based on androgen-dependent responses, which shows a linear association with the Gleason score, metastasis progression and progression-free survival. Our data show that metastatic PCa PDXs, which differ in androgen sensitivity, trigger differential stroma responses, which show the metastasis risk stratification and prognostic biomarker potential.

## 1. Introduction

Bone metastases are detected in 10% of patients already at the initial diagnosis of prostate cancer (PCa) or will develop in 20–30% of the patients subjected to radical prostatectomy and androgen deprivation therapy and will progress to an advanced disease called castration-resistant prostate cancer [1]. Metastases are established when disseminated cancer cells colonize a secondary organ site. An important component of tumor growth is the supportive stroma: the extracellular matrix (ECM) and the nontumoral cells of the matrix microenvironment (e.g., endothelial cells, smooth muscle cells and cancer-associated fibroblasts). Upon interaction of the stroma compartment and tumor cells, the stroma responds by the secretion of growth factors, proteases and chemokines, thereby facilitating the remodeling of the ECM and, thus, tumor cell migration and invasion [2]. Therefore, tumor cell establishment requires an abnormal microenvironment. It is unclear whether the stroma is modulated by the tumor cells or by intrinsic gene expression alterations. Understanding the mechanisms of tumor progression to the metastatic stage is necessary for the design of therapeutic and prognostic schemes.

The bone microenvironment is favorable for the growth of PCa, as well as breast cancer, indicated by the high frequency of bone metastasis in these tumors. Studies have shown that cancer cell growth competes for the hematopoietic niche in the bone marrow with the normal residing stem cells [3], and depending on the cancer cell phenotype, this may lead to either osteoblastic or osteolytic lesions. The stroma signature of osteolytic PCa cells (PC-3) xenografted intraosseously in immunocompromised mice induce a vascular/axon guidance signature [4]. The stroma signature of osteoblastic lesions from human VCap and C4-2B PCa cell lines indicated an enrichment of the hematopoietic and prostate epithelial stem cell niche. A curated prostate-specific bone metastasis signature (Ob-BMST) implicated seven highly upregulated genes (*Aspn*, *Pdgrfb*, *Postn*, *Sparcl1*, *Mcam*, *Fscn1* and *Pmepa1)* [5], among which, *Postn* and *Fscn1* are bone-specific. Furthermore, *Aspn* and *Postn* expression is also increased in primary PCa cases [5], indicative of osteomimicry processes. The induction of osteoblastic genes in the stroma of primary tumors (PCa and breast), such as osteopontin and osteocalcin, has been suggested as a mechanism termed osteomimicry [6] to explain why the bone microenvironment is the preferential metastasis site. High stromal differences between benign, indolent and lethal PCa, combined with the enrichment of bone remodeling genes in high Gleason score cases [7], suggest that the stroma is an active player in PCa. During androgen deprivation, androgen-dependent epithelial cells will undergo apoptosis, while the supporting stroma is largely maintained or replaces the necrotic tissue areas [8]. Stromal cells do express androgen receptors (AR) and have active downstream signaling, while the absence of stromal AR expression is used as a prognostic factor of disease progression [9]. Furthermore, AR binds to different genomic sites in prostate fibroblasts compared to the epithelium [10] and to cancer-associated fibroblasts (CAFs) [11], indicating different roles of AR in epithelial or stroma cellular contexts. Prostate CAFs have tumor-promoting effects on marginally tumorigenic cells (LNCaP), irreversibly altering their phenotype and influencing their progression to androgen independence and metastasis [12,13].

In this study, we investigated whether metastatic PCa patient-derived xenograft models (PDXs) that differ in androgen sensitivity are triggering a differential stroma response. To elucidate the mechanisms of stroma contribution to tumor growth later on, we determined the unique gene expression profile of the stroma compared to the tumor compartment, the proteome changes of the tumor versus stroma. We identified androgen-dependent stroma gene expression signatures with potential disease progression prognostic values for primary PCa.

## 2. Results

### 2.1. Simultaneous Transcriptome Analysis of Human and Murine Signatures in PDXs Can Distinguish Androgen-Dependent Expression Changes in Tumor and Host-Derived Stroma

**Figure 1:**
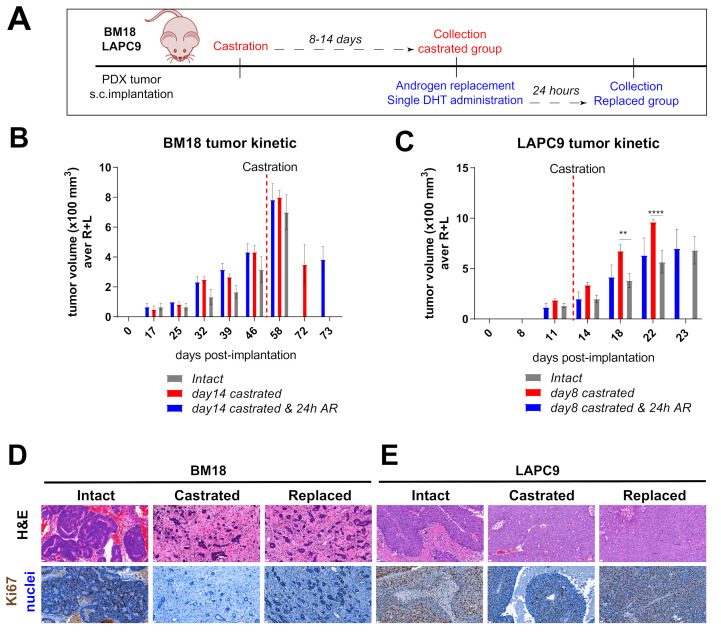
In vivo tumor growth properties of androgen-dependent BM18 versus androgen-independent patient-derived xenograft (PDX) models. (**A**) Scheme of in vivo BM18 and LAPC9 experiments, including the timeline of castration, androgen replacement (single dihydrotestosterone (DHT) administration) and collection of material for transcriptomic analysis. (**B**) BM18 PDX tumor growth progression in time. Groups: (1) intact tumors (collected at max size, *n* = 3), (2) castrated (day 14, *n* = 4) and (3) castrated, followed by testosterone readministration (castrated-testosterone) (day 15 since castration and 24 h since the androgen receptor (AR), *n* = 3). R; right tumor, L; left tumor per animal. (**C**) LAPC9 PDX tumor growth progression in time. Groups: (1) intact tumors (collected at max size, *n* = 3), (2) castrated (day 8, *n* = 4) and (3) castrated, followed by testosterone readministration (castrated-testosterone) (day 9 since castration and 24 h since AR, *n* = 3). Tumor scoring was performed weekly by routine palpation; values represent average calculations of the tumors of all animals per group (considering 2 tumors, left, L, and right, R, of each animal). Error bars represent SEM, calculated considering the no. of animals for each time point. Ordinary two-way ANOVA with Tukey’s multiple comparison correction was performed, *p* ˂ 0.01 (**) and *p* ˂ 0.0001 (****). (**D**) Histological morphology of BM18 and (**E**) LAPC9 (from intact, castrated and androgen-replaced hosts), as assessed by Hematoxylin and Eosin staining (H&E, top). Scale bars: 20 μm, and proliferation marker Ki67 protein expression (bottom panel).

**Figure 2:**
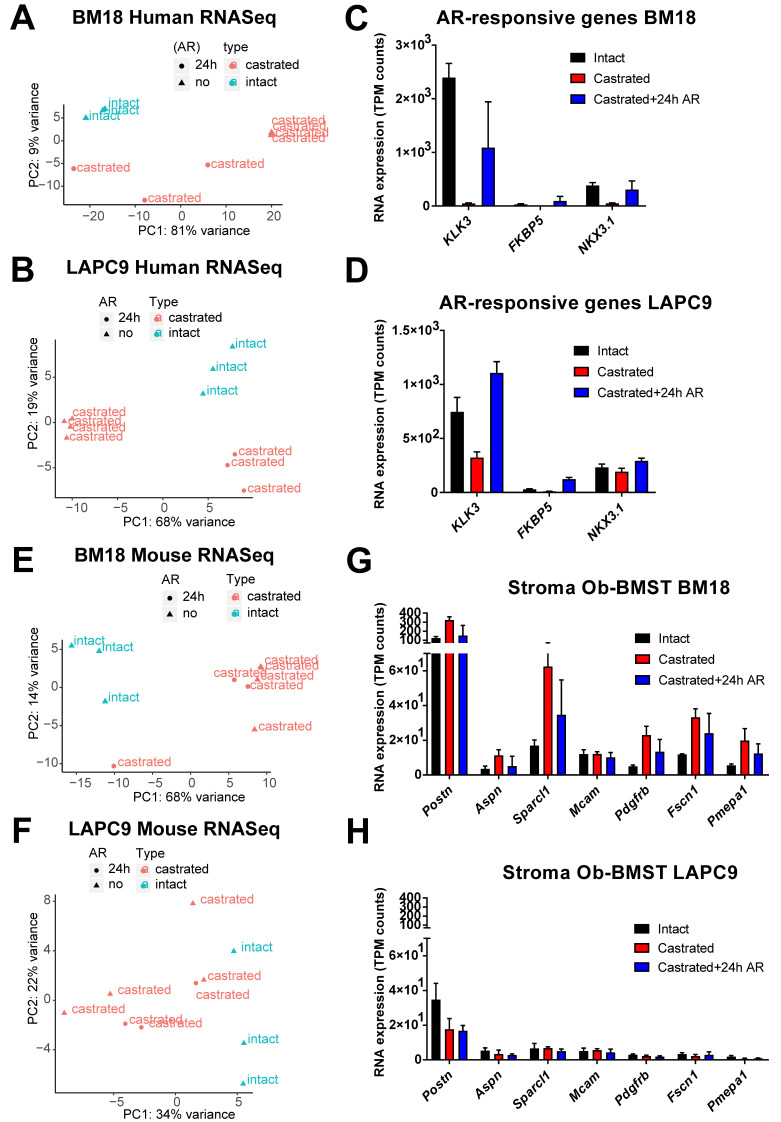
Separation of human (tumor) and mouse (stroma) transcriptomes of BM18 and LAPC9 tumors. (**A**,**B**) Principal component analysis plot of the gene expression of the 500 most variable genes on all samples; BM18 human transcripts (**A**) and LAPC9 human (**B**) at intact, castrated and replaced (castrated + 24 h AR) conditions. (**C**,**D**) Expression values of AR direct target genes as detected by RNA-Seq (transcript per million (TPM) counts) in the BM18 (**C**) or LAPC9 (**D**) tumors as confirmation of the effective repression of AR downstream signaling by castration. Intact (*n* = 3), castrated (*n* = 4) and replaced (*n* = 3). (**E,F**) Principal component analysis plot of the gene expression of the 500 most variable genes on all samples, BM18 mouse (**E**) and LAPC9 mouse (**F**) at intact, castrated and replaced (castrated + 24 h AR) conditions. (**G**,**H**) Expression values of the prostate-specific bone metastasis signature (Ob-BMST) seven upregulated stroma signature genes, as detected by RNA-Seq (TPM normalized counts) in the mouse transcriptome of BM18 (**E**) or LAPC9 (**F**) tumors.

We analyzed the transcriptome of bulk PDX tumors grown subcutaneously in immunocompromised murine hosts by next-generation RNA-sequencing (RNA-Seq). Bone metastasis (BM)18 and LAPC9 PDXs were used in three different states: intact, post-castration (day 8 LAPCa9 and day 14 BM18) and androgen replacement (24 h) (Figure 1A). Tumor growth kinetics revealed the androgen-dependent phenotype of BM18, which regressed completely in two weeks post-castration (Figure 1B), and the androgen-independent phenotype of LAPC9 PDX tumors, which grew exponentially even after castration (Figure 1C), thus confirming the differential aggressiveness of the two models. The reduction of epithelial glands and proliferating Ki67+ cells in the BM18 castrated conditions (Figure 1D) was in contrast to the LAPC9 tumors (Figure 1E), which were morphologically indistinguishable among intact and castrated hosts. Bulk tumor tissues, which contain human tumor cells and mouse infiltrating stroma cells, were simultaneously analyzed from the same samples by RNA-Seq. To distinguish the transcriptome of the different organisms, the mouse and human reads were separated by alignment to a mouse and a human reference genome, respectively. Principal component analysis (PCA) of the human (tumor) 500 most variable genes showed that both castrated and replaced groups have altered expression profiles among each other and compared to the intact tumors. This was the case for the BM18 (Figure 2A) and the LAPC9 human transcriptomes (Figure 2B). The response to short-term androgen replacement showed a larger degree of variability in the BM18 (Figure 2A). However, the expression levels of direct AR target genes (*KLK3, NKX3.1* and *FKBP5*) identified by the RNA-Seq confirmed that androgen levels affected the activation of androgen receptor signaling in both BM18 (Figure 2C) and LAPC9 (Figure 2D, *KLK3* and *NKX3.1*). Differential expression analysis of the most variable human (tumor) genes, showed high variability among the castrated and intact groups, for both BM18 (Figure S1A) and LAPC9 (Figure S1B) transcript levels, while the LAPC9 replaced and castrated groups had similar profile among each other, discriminating them from the intact condition (Figure S1B). 

PCA analysis of the BM18 mouse (stroma) transcriptome indicated that the majority of castrated samples (with and without 24-h androgen replacement) diverged from the intact tumor (Figure 2E). The LAPC9 mouse (stroma) transcriptome instead did not show specific clustering within or between the sample groups when plotting the top 500 most variably expressed genes (Figure 2F). The Ob-BMST signature of all seven genes (*Aspn*, *Pdgrfb*, *Postn*, *Aspn*, *Sparcl1*, *Mcam*, *Fscn1* and *Pmepa1),* which were upregulated in the bone stroma, as previously identified [5], were indeed expressed in the primary PCa TCGA cohort, as well as in both BM18 and LAPC9 PDXs (Figure S2A). *Pdgrfb*, *Postn*, *Aspn* and Sparcl1, specifically in the mouse RNA-Seq data, thus, are stroma-specific. Collectively, the Ob-BMST gene signature is expressed at equal levels in the BM18 and LAPC9 (intact) (Figure S2A). Some of these genes were differentially expressed upon castration in the BM18 (Figure 2G) but not in the LAPC9 (Figure 2H). A bone microenvironment-specific stroma signature induced by osteoblastic cell lines was conserved in bone metastasis PDXs maintained in other microenvironments and found in primary prostatic tissues. 

### 2.2. Proteomic Analysis Provides Functional Information over the Identified Human/Mouse-Specific Transcriptome

**Figure 3:**
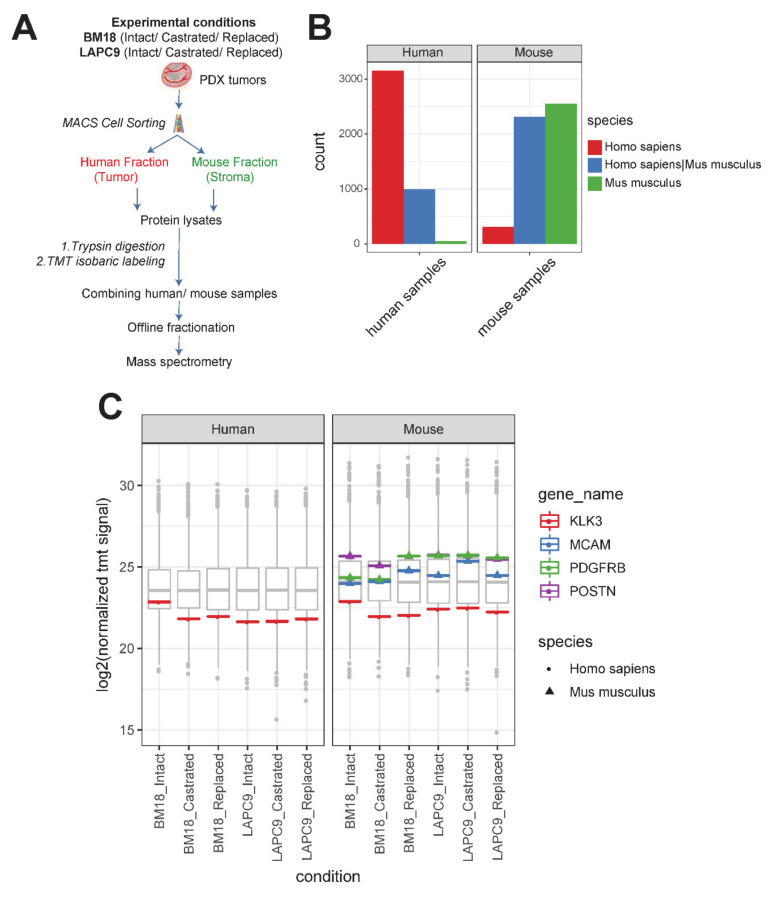
Proteomic analysis of human (tumor) versus mouse (stroma) of BM18 and LAPC9 tumors. (**A**) Experimental separation of human from mouse cell suspensions from fresh tumor isolations by MACS mouse depletion sorting. Cell fractions from intact/replaced (*n* = 3 each), castrated (*n* = 4) biological replicates were pooled into a single replicate (*n* = 1) to achieve an adequate cell number for the proteomic analysis (1 × 10^6^ cells). Protein lysates from the different fractions of BM18/LAPC9 (intact, castrated and replaced) were subjected to Tandem Mass Tag (TMT) labeling (all-mouse or all-human samples were multiplexed in one TMT experiment each), followed by mass spectrometry. (**B**) Detected peptides from human and mouse fractions were searched against a combined human and mouse protein database. Number of species specific or shared proteins is indicated in different colors. (**C**) KLK3 (PSA; Prostate Serum Antigen) protein levels (log_2_ normalized TMT signal sum values) in human cell isolations (left) and in mouse cell isolations (right), and the protein sequence was predicted as human-specific (spheres indicate Homo Sapiens sequence). Seven-up Ob-BMST signature markers POSTN, PDGFRB and MCAM protein levels were absent in human cell isolations (left) and present in mouse cell isolations (right), while all the protein sequences were mouse-specific (triangles indicate Mus Musculus sequences).

To study the proteome of the tumor versus the stroma, human and mouse cell fractions were isolated by the magnetic cell sorting (MACS) mouse depletion method from tumor sample preparations: BM18 and LAPC9 each at the intact, castrated and replaced states. Protein lysates of either mouse or human origins (single replicate from a pool of *n* = 3 to 4 biological replicates per condition) were subjected to an in-solution tryptic digest following Tandem Mass Tag (TMT)-labeling of the resulting peptides and their mass spectrometric analysis (Figure 3A). 

In addition to the initial experimental separation of the protein lysates, we further explored the species homologs of the identified proteins by computational analysis using a combined human and mouse protein sequence database. We identified 4198 proteins in the sample that were enriched for human cells. Thereof, 3154 were human-specific proteins, with 996 revealing a high homology shared among human and mouse, and only a fraction of 48 mouse-specific, peptides. (Figure 3B, left plot). For samples enriched in mouse cells, we identified, in total, 5192 proteins; thereof, 2486 mouse-specific proteins, 2379 shared homologs and 247 human-specific (Figure 3B, right plot). We searched for prostate specific markers such as KLK3, a prostate-specific antigen that is secreted by luminal cells. In the proteomic data, the human-specificity was confirmed, and the secreted protein was found also in the mouse fraction (Figure 3C). To further ensure that the proteomic data were indeed identifying real stromal-specific candidates, we searched specifically for the seven-gene Ob-BMST signature found also to be expressed in both BM18 and LAPC9. POSTN, PDGFRB and MCAM (Figure 3C) were indeed detected at the protein level, thus might have a functional role, and were found exclusively in the mouse fraction (Figure 3C, right plot) and hybridizing with mouse-specific sequences (Figure 3C, triangle indicates Mus Musculus species specificity). 

### 2.3. Differential Expression Analysis Reveals Androgen-Dependent Stromal Gene Modulation in Androgen-Independent PDX Model

**Figure 4:**
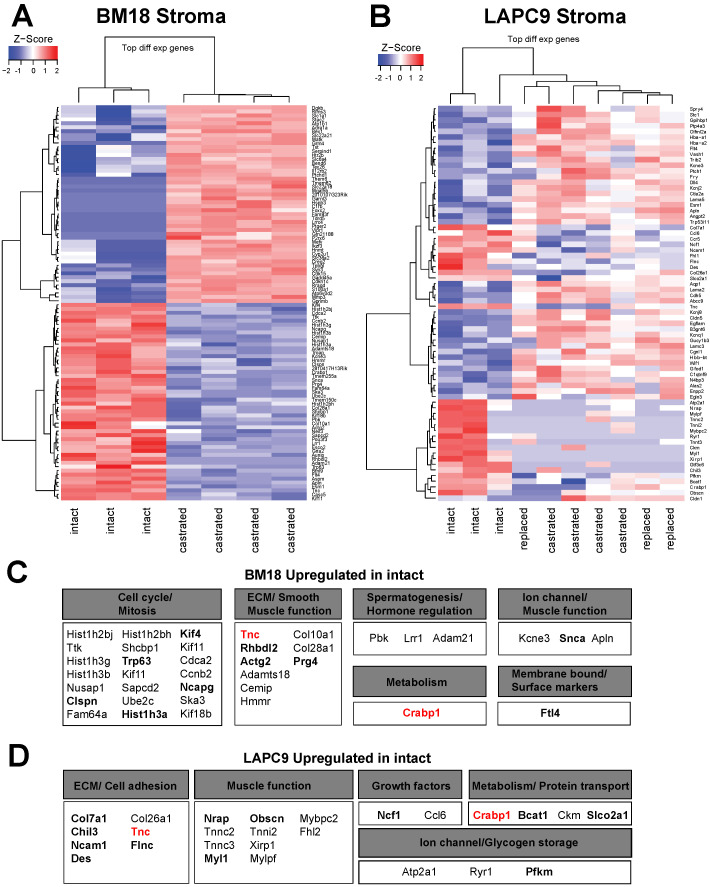
Differential expression analysis of the transcriptome indicates different expression profiles of stromal genes as response to androgen deprivation. (**A**) Heatmap represents a differential expression analysis of the most variable genes from the mouse transcriptome of BM18 castrated compared to BM18 intact tumors. Genes modulated by androgen deprivation due to castration in the up/downregulation compared to intact tumors are indicated in red or blue colors, respectively. (**B**) Heatmap represents Z-score of the differential expression analysis of most variable genes in the mouse transcriptome of LAPC9 castrated (with and without androgen replacement) compared to LAPC9 intact tumors. (**C**) Description of mouse genes found upregulated in BM18 intact tumors and the biological processes they are involved in, according to the Gene Ontology (GO) terms. (**D**) Description of the mouse genes found upregulated in LAPC9 intact tumors and the biological processes they are involved in, according to the GO terms.

The relative ratio of human and mouse transcript reads reflected a higher stroma content in the BM18 compared to LAPC9 and significantly reduced human tumor content with enriched stroma content in the BM18 castrated group (Figure S2B). No major differences were observed in the LAPC9 castrated group (Figure S2C). We demonstrated that the human (tumor), as well as the mouse (stroma), transcriptomes follow androgen-dependent transcriptomic changes in the BM18 groups (intact versus castrated versus replaced) (Figure 2A,E). Venn Euler diagrams illustrate androgen level-dependent stromal gene expression modulation not only in the BM18 (Figure S2D and Table S1) but, also, in the androgen-independent (in terms of tumor growth) LAPC9 model (Figure S2E,F and Table S1). To identify the top-most significant AR-regulated stromal genes, we performed a differential expression analysis of BM18 tumors (Figure 4A) from castrated hosts and compared it to BM18 intact (the replaced tumors were not included here due to higher variability). Of the top-most variable genes, 50 were highly upregulated in BM18 tumors (z-score >1) and downregulated upon castration (Figure 4A). A differential expression analysis of LAPC9 tumors from castrated/replaced tumors versus intact tumors revealed the top-most differentially regulated genes: the 27 most upregulated genes in intact, which were downregulated in the castrated groups (Figure 4B). Among the 50 mouse genes that were highly upregulated in the intact BM18, and significantly modulated by castration, were 23 genes implicated in cell cycle/mitosis, 10 implicated in ECM and 3 related to spermatogenesis/hormone regulation, according to the Gene Ontology terms (Figure 4C). Two of these genes, *Tnc* and *Crabp1*, were also detected in the proteomic data (Figure 4C, highlighted in bold) and in both PDXs (Figure 4C,D, highlighted in red). Among the 27 mouse genes that were highly upregulated in the intact LAPC9, and significantly modulated by castration, seven genes were implicated in ECM/cell adhesion/smooth muscle function, and 14 were implicated in non-smooth muscle function and metabolism based on the Gene Ontology terms (Figure 4D). In the LAPC9 proteomic data, we detected 14 genes out of the 27 to be expressed in the mouse fractions (Figure 4D, bold), indicative of potential functional values. Of interest in potentially mediating tumor stroma extracellular interactions are a neural adhesion protein (*CD56*), implicated in cell–cell adhesion and migration by homotypic signaling, as well as Tenascin C (*Tnc*), an extracellular protein that is found abundantly in the reactive stroma of various cancer types, yet not expressed in normal stroma. Both genes were expressed at the protein level, exclusively in the mouse compartment of the BM18 and LAPC9, at all states (intact, castrated and replaced). Furthermore, Tnc was detected in both BM18 and LAPC9 at the transcriptional and proteomic levels and was reactivated after 24 h of androgen replacement (Figure 4B), indicative of AR-direct target gene modulation.

### 2.4. Cross Comparison of Stromal Transcriptome among Different PDXs Identifies ECM and Cell Adhesion Pathways in the LAPC9 Androgen-Independent Model

**Figure 5:**
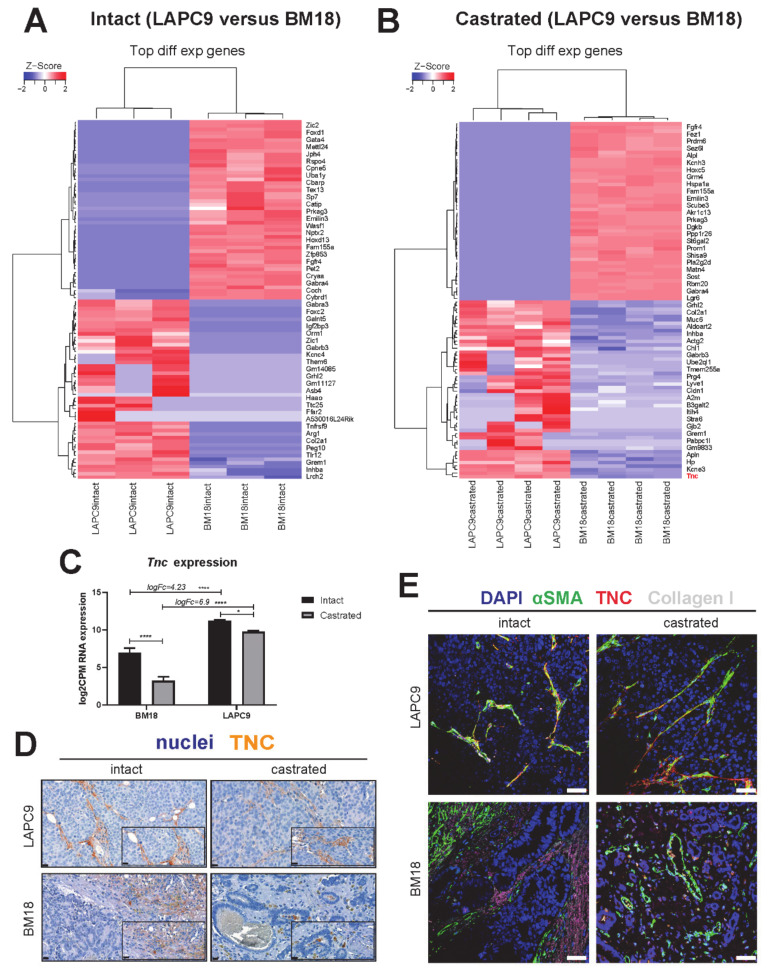
Cross-comparison of LAPC9 versus BM18 suggests stromal gene Tenascin C expression being associated with advanced PCa and regulated by androgen levels. (**A**) Heatmap represents the differential expression analysis of the top 100 most variable genes from the mouse transcriptome of LAPC9 intact tumors compared to BM18 intact tumors and (**B**) of LAPC9 castrated tumors compared to BM18 castrated tumors. (**A**) Subset of genes in LAPC9 samples have zero counts, leading to the same z-score, while the same genes are highly expressed in BM18 samples. (**C**) *Tnc* RNA expression (log_2_CMP counts) in the stroma transcriptome. Log_FC_ (fold change) enrichment of *Tnc* in LAPC9 over BM18 is indicated. Ordinary two-way ANOVA with Tukey’s multiple comparison correction was performed, *p* ˂ 0.05 (*) and *p* ˂ 0.0001 (****). (**D**) Tenascin protein expression and stromal specificity assessed by immunohistochemistry in LAPC9 and BM18 tumors, both at the intact and castrated states. Scale bars: 20 μm. (**E**) Tenascin protein (indicated in red) colocalization with stromal markers, smooth muscle actin (αSMA, green) and collagen type I (gray) assessed by immunofluorescence in LAPC9 and BM18 tumors, both at the intact and castrated states. DAPI marks the nuclei. Scale bars: 50 μm.

To assess the similarity between the stromal transcriptome of the androgen-independent LAPC9 and the BM18, a differential expression analysis was performed. In a panel of the 50 top-most variable genes comparing the tumors at their intact conditions, we identified several genes that follow the same pattern of modulation in intact tumors (Figure 5A) and in castrated tumors (Figure 5B). Of interest were the ECM-related genes downregulated in LAPC9 versus BM18; the Fibroblast Growth Factor receptor (*Fgfr4*), elastin microfibril interface (*Emilin3*) and upregulated collagen type 2 chain a1 (*Col2a1*). 

The differential expression of LAPC9 castrated versus BM18 castrated highlighted genes that were identified in the analysis among LAPC9 castrated, replaced versus LAPC9 intact, such as Apelin (*Apln*), *Col2a1* and Tenascin C (*Tnc*). 

To identify the biological processes ongoing in the LAPC9 compared to BM18, a pathway analysis was performed on the differentially expressed murine genes of the LAPC9 versus the BM18. Enrichment maps of the top 20 enriched GO biological pathways highly overlap pathways, such as ECM, focal adhesion and cell adhesion/migration in the intact and castrated LAPC9 (Figure S3A,B). Similarly, among the KEGG pathway sets, there was an enrichment of stroma regulation (e.g., actin cytoskeleton, focal adhesion and cell adhesion) and bone and immune-related processes (e.g., osteoclast differentiation) (Figure S3C–F and Table S2). The enrichment of cancer-related pathways (e.g., PI3K/AKT, proteoglycans in cancer, pathways in cancer) was commonly found in the LAPC9 intact and castrated stroma transcriptomes (Figure S3E,F and Table S2).

Given that genes activated in a castrated state might be indicative of androgen resistance mechanism activation, we postulated that genes upregulated in the androgen-resistant LAPC9 over the androgen-dependent BM18 might be relevant for understanding the aggressive phenotype of LAPC9 and, therefore, of the advanced metastatic phenotype of similar tumors. One of those genes, Tenascin, is an ECM protein that is produced at the (myo)fibroblasts that is virtually absent in normal stroma in the prostate and other tissues and has been associated with the cancerous reactive stroma response in different cancers. We interrogated the expression of *Tnc* in the RNA-Seq data and found that it was highly upregulated in LAPC9 compared to BM18 both in intact (log_FC_ 4.23, *p* ˂ 0.001) and among the castrated conditions (log_FC_ 6.9, *p* ˂ 0.001) (Figure 5C). However, in both models, the *Tnc* levels significantly decreased upon castration (BM18, *p* ˂ 0.001 and LAPC9, *p* ˂ 0.05), indicating the potentially AR-mediated regulation of *Tnc* expression. In LAPC9 tumors, the TNC protein is expressed in the tumor-adjacent ECM and in the proximity of vessels (Figure 5D, intact and castrated) and co-expressed by smooth muscle actin (αSMA)- and collagen type I-positive myofibroblasts (Figure 5E). Instead, the intact BM18 tumors show TNC and collagen type I deposition in the ECM, but there is no overlap with αSMA-positive myofibroblasts (Figure 5D,E, BM18 intact). Castrated BM18 tumors have minimal TNC expression, found only in cells proximal to the remaining epithelial glands, yet with no typical fibroblast/stromal morphology (Figure 5D,E, BM18 intact), suggesting an altered phenotype of TNC upon androgen deprivation. 

### 2.5. Protein Expression of Tenascin and Its Interaction Partners 

**Figure 6:**
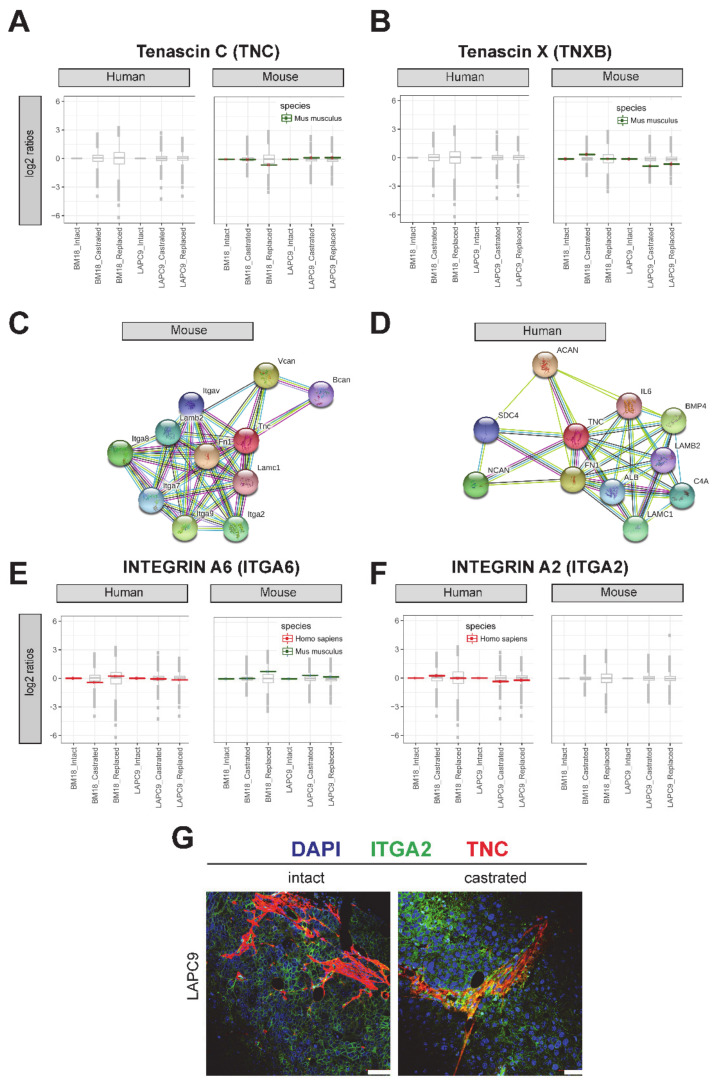
Tenascin C and its predicted interaction partners analyzed by mass spectrometry. (**A**) Tenascin C (TNC) and (**B**) alternative isoform Tenascin X (TNXB) protein relative abundance (log_2_ ratios; single replicates per sample from a pool of *n* = 3 to 4) in human cell isolations (left) and present in mouse cell isolations (right). The variance stabilization normalization (vsn)-corrected TMT reporter ion signals were normalized by the intact conditions of either BM18 or LAPC9. The protein sequences were predicted as mouse-specific (green). (**C**) Protein interaction network of the mouse TNC protein based on the STRING association network (https://string-db.org/). (**D**) Protein interaction network of the human TNC protein based on the STRING association network https://string-db.org/. (**E**) Predicted TNC-binding partner integrin A6 (ITGA6) was detected by mass spectrometry in both the human and mouse protein lysates and matching the organism-specific protein sequence based on the bioinformatics analysis (red for human and green for mouse). (**F**) Predicted TNC-binding partner integrin A2 (ITGA2) was detected by mass spectrometry, specifically in the human protein lysates, and matched the human-specific protein sequence. (**G**) Spatial localization of the Tenascin protein (TNC, indicated in red) and integrin A2 (ITGA2, green) assessed by immunofluorescent co-labeling in LAPC9 intact and castrated tumors. DAPI marks the nuclei. Scale bars: 50 μm.

To assess whether the transcriptomic changes of *Tnc* in the PDX models corresponds to the functional protein and, thus, a relevant role in bone metastatic PCa, we performed a proteomic analysis. A mass spectrometry analysis of the human and mouse fractions indicated that the Tnc protein was expressed specifically in the mouse (stromal) fractions in BM18 and LAPC9 (Figure 6A). The isoform Tenascin X was also expressed at the protein level (Figure 6B). The interaction network of the mouse protein Tnc is based on experimental observations and prediction tools (STRING) and consists of laminins (Lamc1 and Lamb2); fibronectin (Fb1); integrins (Itga2, a7, a8 and a9) and proteoglycans (Bcan and Vcan) (Figure 6C). The human interactome is less-characterized, yet most of the interactome is conserved: laminins (LAMC1 and LAMB2); proteoglycans (NCAN and ACAN) and others such as interleukin 8 (IL-8), BMP4, ALB and SDC4 (Figure 6D). However, integrin interaction-binding partners in a human setting have not been confirmed. Given the importance of integrins for cell adhesion and migration known to be found in mesenchymal/stromal and epithelial tumor cells, we focused on the expression of human- and mouse-derived integrins. The *ITGA9*, *ITGA6* and *ITGA2* were all found to be expressed in both the RNA-Seq and proteomic data (Figure 6E, ITGA6, respectively); however, only the *ITGA2* protein was specifically found in the human counterpart and not overlapping with the mouse stroma (Figure 6F). Co-labeling both proteins indicated adjacent spatial localization with TNC deposition in close proximity to ITGA2-positive epithelial cells (Figure 6G); however, whether those cell populations acquired different properties compared to other epithelial cells has yet to be investigated. Overall, the tumor *ITGA2* and stromal *Tnc* is a potential molecular interaction, possibly part of the dual cellular communication among a tumor and its microenvironment cellular types and ECM. 

### 2.6. Stromal Tenascin Expression as a Prognostic Factor of Disease Progression in High-Risk PCa

**Figure 7:**
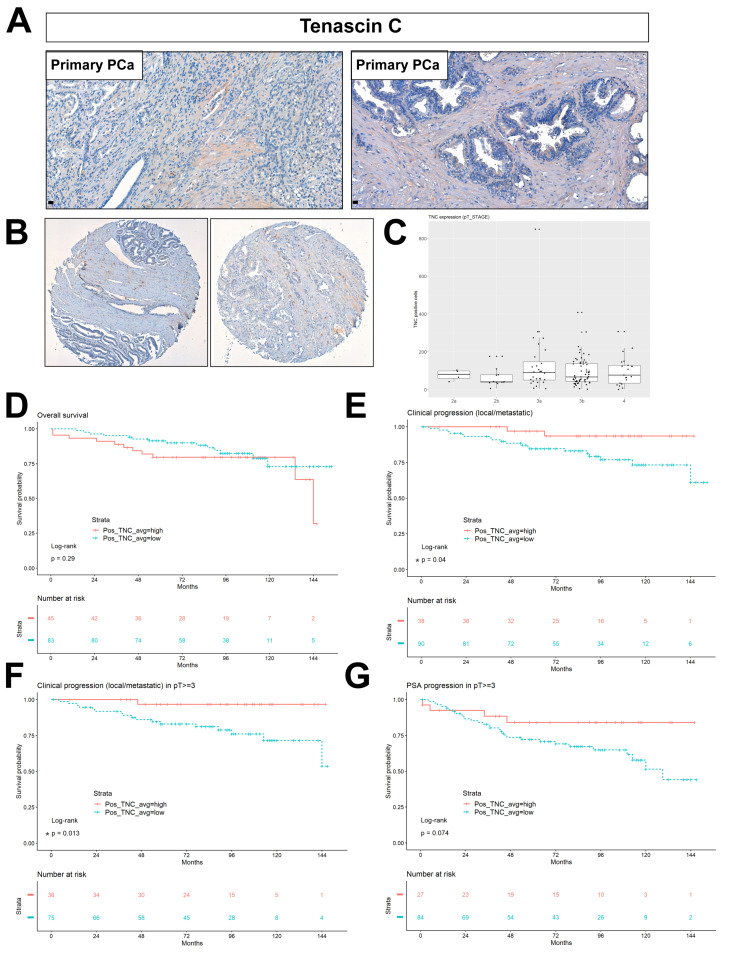
TNC protein expression is a negative metastasis prognostic factor in primary, high-risk PCa. (**A**) Validation of the protein expression and stromal specificity of TNC by immunohistochemistry in primary PCa cases. (**B**) Representative cases of TNC staining on primary PCa Tissue Microarray (TMA) from European Multicenter Prostate Cancer Clinical and Translational Research Group (EMPaCT). (**C**) TNC expression levels in terms of the no. of positive cells in the pT Stage classification. Statistical multiple comparison test, the Wilcoxon rank sum test, was performed; *p* > 0.05 (**D**) Overall survival probability in patient groups of TNC-high and TNC-low (no. of positive, TNC-expressing cells) (*p* = 0.29, ns—non significant). Average value represents the mean of four cores per patient case. (**E**) Clinical progression to the local recurrence or metastasis probability in patient groups of TNC-high and TNC-low expressions (*p* = 0.04 and * ˂ 0.05). (**F**) Clinical progression to the local recurrence or metastasis probability among patients of pT Stages 3a, 3b and 4 based on TNC-high and TNC-low expressions (*p* = 0.013 and * ˂ 0.05). (**G**) PSA progression probability among patients of pT Stages 3a, 3b and 4 based on the TNC-high and TNC-low expressions (*p* = 0.074, ns).

**Figure 8:**
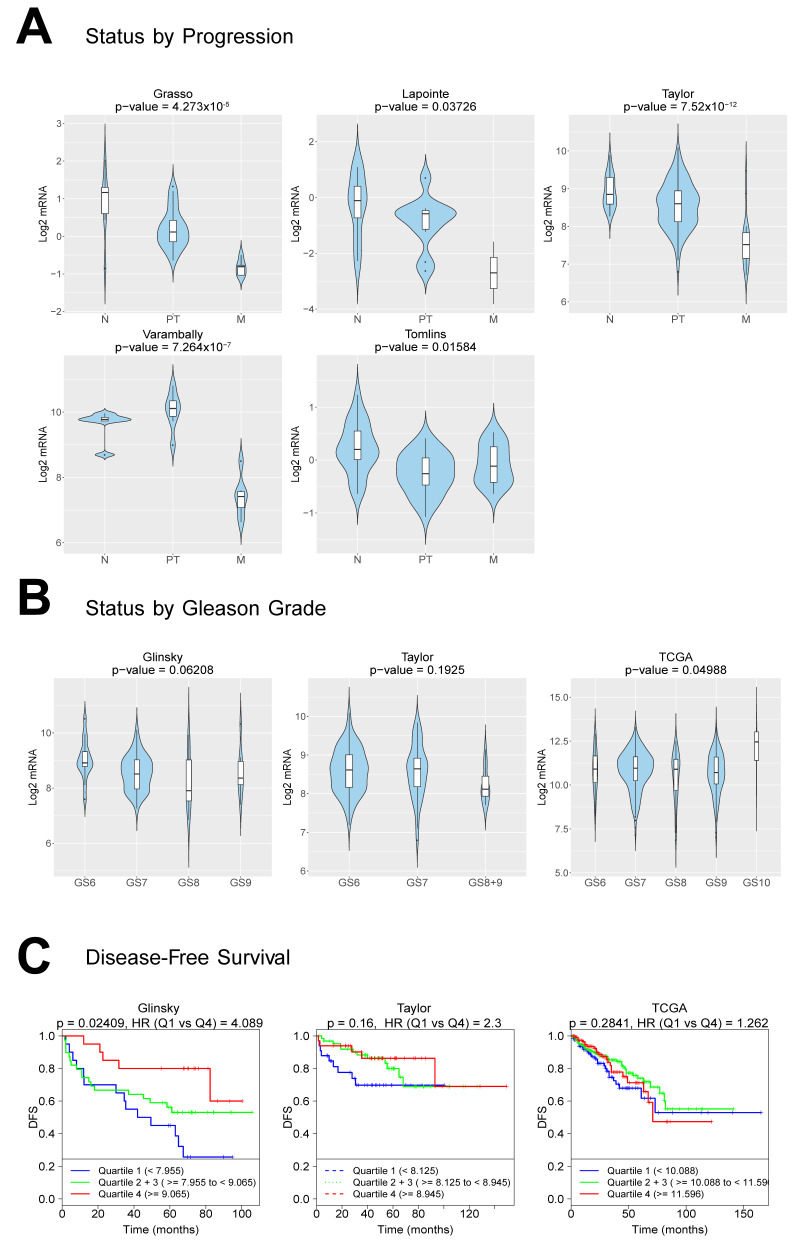
*TNC* RNA expression is inversely correlated with the disease progression, Gleason score and survival. (**A**) Violin plots depicting the expression of *TNC* among nontumoral (N), primary tumor (PT) and metastatic (M) PCa specimens in the indicated datasets. The Y-axis represents the Log_2_-normalized gene expression (fluorescence intensity values for microarray data or sequencing read values obtained after gene quantification with RNA-Seq Expectation Maximization (RSEM) and normalization using the upper quartile in case of RNA-seq). An ANOVA test is performed in order to compare the mean gene expression among two groups (nonadjusted *p*-value), obtained by a CANCERTOOL analysis. (**B**) Violin plots depicting the expression of *TNC* among PCa specimens of the indicated Gleason grade in the indicated datasets. The Gleason grades are indicated as GS6, GS7, GS8, GS8+9, GS9 and GS10. An ANOVA test is performed in order to compare the mean among groups (nonadjusted *p*-value), obtained by a CANCERTOOL analysis. (**C**) Kaplan-Meier curves representing the disease-free survival (DFS) of patient groups selected according to the quartile expression of *TNC*. Quartiles represent ranges of expression that divide the set of values into quarters. Quartile color code: Q1 (Blue), Q2 plus Q3 (Green) and Q4 (Red). Each curve represents the percentage (Y-axis) of the population that exhibits a recurrence of the disease along the time (X-axis, in months) for a given gene expression distribution quartile. Vertical ticks indicate censored patients. Quartile color code: Q1 (Blue), Q2 plus Q3 (Green) and Q4 (Red). A Mantel-Cox test is performed in order to compare the differences between curves, while a Cox proportional hazards regression model is performed to calculate the hazard ratio (HR) between the indicated groups. Nonadjusted *p*-values are shown. Analysis obtained by CANCERTOOL.

The detection of key mouse stromal genes in PCa PDXs gives the opportunity to evaluate the role and potential prognostic value of the human orthologs of these stromal genes. To validate the localization and stromal specificity of TNC protein expression, we performed immunohistochemistry on the primary PCa tissue sections. TNC is localized in the extracellular space (Figure 7A, primary cases). Next, we evaluated the TNC expression in a tissue microarray of 210 primary prostate tissues, part of the European Multicenter High Risk Prostate Cancer Clinical and Translational research group (EMPaCT) [14,15,16] (Figure 7B–G). Based on the preoperative clinical parameters of the TMA patient cases (Table 1, Table 2) and the D’Amico classification system [17], they represent intermediate (clinical T2b or Gleason *n* = 7 and PSA >10 and ≤ 20) and high-risk (clinical T2c-3a or Gleason score (GS) = 8 and PSA ≥ 20) PCa. The number of TNC-positive cells (Figure 7B) were quantified and averaged for all cores (four cores per patient case) in an automated way, including tissue selection, core annotation and equal staining parameters set. To investigate the association between the number of TNC-positive cells and patient survival or disease progression, we calculated the optimal cut-point for the number of TNC-positive cells by estimation of the maximally selected rank statistics [18]. Association between TNC-expressing cells and pT Stage indicated that the majority of cases cluster towards stages 3a and 3b (Figure 7C). A multiple comparison test among all groups showed no statistically significant association between the TNC expression and pathological stage (Table S3**,** *p* > 0.05). The overall survival probability between two patient groups with, respectively, high and low numbers of TNC-positive cells was indifferent (*p* = 0.29, Log-rank test) (Figure 7D). We focused on the probability of TNC expression in primary tumors to be a deterministic factor for clinical progression to local or metastasis recurrence. Clinical progression probability was higher in the TNC-low group compared the TNC-high group (*p* = 0.04 *, Log-rank test) (Figure 7E). Next, we examined the clinical progression in patients with pT Stage ≥3 (groups 3a, 3b and 4). The high T-Stage cases did separate into two groups based on the TNC expression, with the TNC low-expressing group exhibiting earlier a clinical progression (local or metastatic recurrence, *p* = 0.013 *, Log-rank test) (Figure 7F). The PSA progression probability in patients with pT Stage ≥3 indicated an association trend of a TNC-low group with earlier biochemical relapse events (*p* = 0.07, Log-rank test) (Figure 7G). Similarly, the TNC-low group correlated with a higher probability for PSA progression after radical prostatectomy among cases with carcinoma-containing (positive) surgical margins (Figure S4A**,** *p* = 0.031 *, Log-rank test) or positive lymph nodes (Figure S4B, *p* = 0.092, Log-rank test). A low number of TNC-expressing cells coincides with a poor prognosis in terms of metastasis progression, similarly to its downregulation upon castration in the bone metastasis PDXs (Figure 4B) based on the RNA-Seq analysis. To further evaluate the clinical relevance of this finding, in multiple clinical cohorts with available transcriptomic data and clinical information, a CANCERTOOL analysis was performed [19]. Similar to the protein TMA data (Figure 7), the *TNC* mRNA levels were significantly downregulated during the disease progression from primary to PCa metastasis, compared to the expression in the normal prostatic tissues in all five datasets tested (Figure 8A). The *TNC* expression shows a pattern of inverse correlations, with the Gleason score among GS6 to GS9; however, it significantly discriminated patient groups for the Gleason score in one out of three datasets tested (Figure 8B, TCGA dataset * *p* = 0.049, Glinsky *p* = 0.06, Taylor *p* = 0.192), with the highest expression found in a high GS10 group and indifferent among GS6-GS9. A disease-free survival analysis indicated that a low TNC expression is associated with a worse prognosis based on the Glinsky dataset (Q1 Glinsky et al. [20], * *p* = 0.02), while no statistically significant association was observed in the Taylor and TCGA dataset (Figure 8C). Overall, the TNC expression in tumor samples, both at the RNA and protein levels, becomes progressively less abundant in primary and metastasis PCa specimens, while a low TNC expression is significantly associated with the disease progression and poor disease-free survival (DFS) outcome. 

### 2.7. Stroma Signatures from Androgen-Dependent and -Independent States Correlate with Disease Progression 

**Figure 9:**
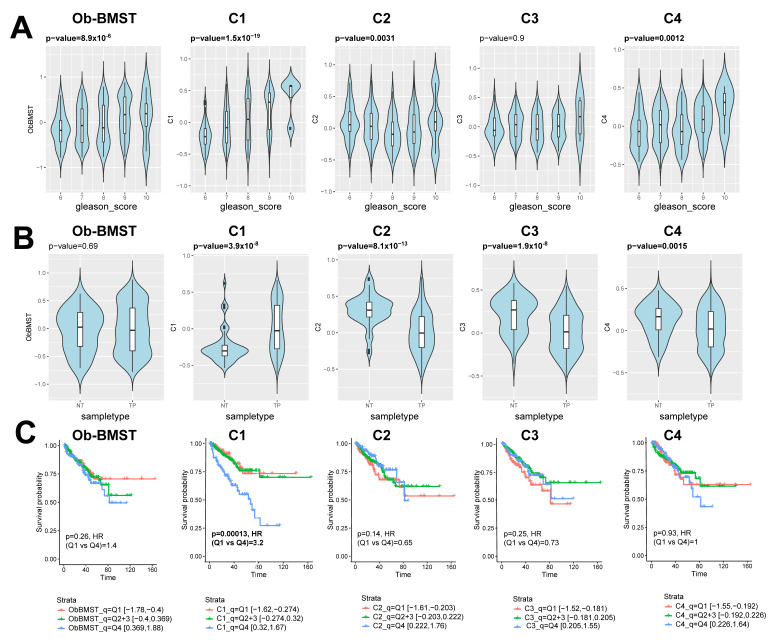
Stroma signatures identified from bone metastatic PDXs as prognostic biomarkers in primary PCa. (**A**) Violin plots showing Gene Set Variation Analysis (GSVA) signature scores of the Ob-BMST, C1-C4 gene sets, stratified by Gleason score from the TCGA cohort. Box-and-whisker plots illustrating median (midline), inter-quartile range (box), with the whiskers extending to at most 1.5 IQR from the box. Outliers beyond the range of the whiskers are illustrated as dots. P-values computed by Spearman correlation tests. (**B**) Violin plots showing GSVA signature scores of the Ob-BMST and C1-C4 gene sets stratified by sample types (NT: nontumor and TP: primary tumor) from the TCGA cohort. Box-and-whisker plots illustrating the median (midline) and interquartile range (box), with the whiskers extending to at most 1.5 IQR from the box. Outliers beyond the range of the whiskers are illustrated as dots. *P*-values computed by Mann-Whiney U tests. (**C**) Kaplan-Meier plots of progression-free survival (PFS) stratified as the bottom 25% (Q1), middle 50% (Q2 and 3) and top 25% (Q4) of the signature scores of the Ob-BMST and C1-C4 gene sets. *P*-values and hazard ratios computed by Cox proportional hazard regression.

In order to comprehensively map the stroma responses related to the disease severity, we analyzed the stroma gene signature lists associated to androgen dependency and aggressive androgen-independent states. The stroma signatures are categorized in clusters (C1–C4, Table S4) based on a differential expression analysis (Figure 4 and Figure 5): C1 (50 highly upregulated genes in BM18 intact that get downregulated upon castration), C2 (27 highly upregulated genes in LAPC9 intact that get downregulated upon castration), C3 (32 highly upregulated genes in LAPC9 intact compared to BM18 intact) and C4 (24 highly upregulated genes in LAPC9 castrated compared to BM18 intact). Clusters C1 and C2 aim to identify the most responsive genes to androgen deprivation. C3 and C4 are designated to identify the genes/pathways enriched in the stroma of castration-resistant prostate cancer (CRPC) compared to the androgen-dependent tumor model. The TNC gene was among the signature list: C1, C3 and C4. The prognostic potential of the C1-C4 signatures in comparison to the bone signature Ob-BMST was tested on the TCGA cohort based on the Gleason score, gene expression and outcome data (Figure 9 and Table S5). The high signature scores of Ob-BMST, C1, C2 and C4 had statistically significant positive correlations with the high GS groups (Figure 9A, Ob-BMST and C1 (*p* ˂ 0.001), C2 and C4 (*p* ˂ 0.01)). In terms of gene expression, the C1 signature was significantly higher in primary tumors versus normal tissues (Figure 9B, *p* ˂ 0.001), while the C2, C3 and C4 have lower signature scores in the tumor samples compared to normal (Figure 9B, C2 and C3 (*p* ˂ 0.001) and C4 (*p* ˂ 0.01)). Kaplan-Meier plots of progression-free survival (PFS) stratified as the bottom 25% (Q1), middle 50% (Q2 and 3) and top 25% (Q4) showed significant correlations among the high signature scores (Q4) of the C1 gene set and PFS (Figure 9C, *p* ˂ 0.001), while none of the other gene lists showed significant correlations. 

To further assess the prognostic performance of the signatures, we correlated the C1-C4 gene signatures with PCa-specific stroma signatures identified by Tyekucheva et al. [7] and Mo et al. [21] (Table S4) across two cohorts containing both primary and metastatic PCa that were used [22,23]. The C3 and C4 showed the strongest linear correlations with the Tyekucheva and the Mo_up (upregulated in metastases) signatures when tested across the Grasso dataset (Figure S5A, r > 0.64), while the C4 signature also had positive correlations when tested across the Taylor et al. dataset (Figure S5B, r > 0.6). The C1 signature did not significantly correlate with the gene lists tested (Figure S5A, C1 *p* > 0.05). The low signature score of the C2 and C3 were significantly associated with metastatic disease progression (Figure S5B, *p* ˂ 0.001) in both cohorts tested, and C4 showed a similar pattern (Figure S5B, C4 *p* = 0.062). A common pattern of the stroma signatures is a similar or enriched signature score at the primary stage compared to benign/normal tissue, and lower/depleted signature scores at the metastasis stage (Figure S5C,D; C2, C3 and C4, Tyekucheva and Mo and Figure 9B; C2-C4). Only a significant correlation with the Gleason score was observed by the C1 signature list, with a high signature score found at the high GS patient groups (Figure S5E, *p* ≤ 0.001), which is in concordance to the linear correlation with metastatic disease in all clinical cohorts tested (Figure S5C,D, *p* ≤ 0.001 and Figure 9, TCGA).

## 3. Discussion

The role of the microenvironment upon cancer formation and progression to metastasis is supported by numerous studies [24,25]; however, the current knowledge is not sufficient to reconstruct the chain events from primary to secondary tumor progression. The normal stroma microenvironment is considered to halt tumor formation; however, after interactions with tumor cells, it also undergoes a certain “transformation” at the transcriptomic, and even at the genetic, levels [26,27,28,29]. The processes by which PCa tumor cells affect stroma and, in turn, stroma impacts primary PCa tumor growth or metastasis are complex and remain largely unclear. 

We utilized well-established bone metastasis PDX models, which can be propagated subcutaneously and have different aggressiveness in terms of androgen dependency: the CRPC model LAPC9 representing complete androgen-independent advanced disease [30] and the BM18 that mimics human luminal PCa [31,32] and uniquely retains androgen sensitivity, typically seen in the primary and treatment-naïve stages. The androgen-independent stem cell populations that survive castration are well characterized in both models [31,33,34]; yet, the contribution of the stroma in those district tumor phenotypes has not been investigated. In vivo PDX models grafted in immunocompromised mice, although they lack the complexity of a complete immune system, represent the stroma compartment (endothelial cells, smooth muscle cells, myofibroblasts and cancer-associated fibroblasts). Due to the subcutaneous growth of BM PCa PDXs, the human stroma is replaced by mouse-infiltrating stromal cells and vasculature [35,36]. Mouse cell infiltration allows the discrimination of organism-specific transcripts, human-derived transcripts representing the tumor cells and mouse-derived transcripts representing the mouse stroma compartment. Using next-generation RNA-Seq, MACS-based human and mouse cell sorting, mass spectrometry and organism-specific reference databases, we have identified the tumor-specific (human) from the stroma-specific (mouse) transcriptomes and proteomes of bone metastasis PCa PDXs. The dynamics of AR signaling in the stroma are best represented in an in vivo setting [11]; therefore, to specifically examine the stroma changes dictated by PCa cells, we subjected the PDXs in androgen and androgen-deprived conditions. By imposing this selection pressure, we could identify androgen-dependent gene expression patterns. 

We demonstrated that the human (tumor), as well as the mouse (stroma), transcriptomes follow androgen-dependent transcriptomic changes in the BM18 groups (intact versus castrated versus replaced). Despite the androgen-independent tumor growth of LAPC9, at the gene expression level, the LAPC9 tumor cells do follow AR-responsive patterns (human transcriptomes). However, the principal component analysis showed that, although castrated and replaced LAPC9 groups separate adequately based on the human transcriptome, they appear to have overall uniform stromal transcriptomes. 

We report that transcriptomic mechanisms linked to osteotropism were conserved in bone metastatic PDXs, even in nonbone environments, and differential stroma gene expressions are induced by different tumors, indicating the tumor specificity of stroma reactivity. The Ob-BMST signature of all seven genes (*Aspn*, *Pdgrfb*, *Postn*, *Sparcl1*, *Mcam*, *Fscn1* and *Pmepa1)*, which were upregulated in bone stroma previously identified [5], were indeed expressed in both BM18 and LAPC9 PDXs, specifically in the mouse RNA-Seq and, also, expressed at the protein level, as identified by mass spectrometry. The gene expression modulation of mouse stroma is, ultimately, an important evidence of the effects of tumor cells in their microenvironment, where they induce favorable conditions for their growth. 

The differential expression analysis of the LAPC9 stroma signature from intact, castrated and replaced hosts highlighted the most significantly variable genes, which were modulated by androgen levels, despite the androgen-independent tumor growth phenotype. Focusing on the genes that were highly activated in intact but strongly modulated by castration, we categorized these genes based on Gene Ontology terms. We found that LAPC9 stromal genes were ECM remodeling components and genes involved in smooth muscle function or even in striated muscle function. Of interest are *CD56*, *Tnc* and *Flnc*. Among the BM18 most abundant stromal transcripts are genes involved in cell cycle regulation and cell division. Interrogating the differences among the two models, we focused on the transcriptome of LAPC9 normalized versus the less aggressive, androgen-dependent BM18. In particular, *Tnc* is expressed in both PDXs, higher in LAPC9, yet downregulated upon castration, suggesting a direct AR gene regulation. The differential expression analysis among both the PDXs after castration indicated that *Tnc* is upregulated more in LAPC9 than BM18, suggesting an association with disease aggressiveness. Genes that become upregulated in castrated conditions are likely to be linked to androgen resistance; thus, we studied *Tnc* for its potential role in metastasis progression. 

TNC is an extracellular glycoprotein absent in normal prostates and postnatally silenced in most tissues. TNC is re-expressed in reactive stroma in human cancers, and there is evidence of its expression in low-grade tumors (Gleason 3) of human PCa [37] and, possibly, already activated at the prostatic intraepithelial neoplasia (PIN) stage [38,39]. In particular, high molecular weight TNC isoforms are expressed in cancer due to alternative mRNA splicing [38]. We examined whether an abundance of TNC-positive cells in primary PCa TMA can predict the metastatic progression and overall survival (12 years follow-up after radical prostatectomy). A high number of TNC-positive cells did not correlate with the overall survival or histological grade, in agreement with previous data [38]. The PSA progression after radical prostatectomy occurred earlier in the TNC-low group compared to the TNC-high group when high stage cases (pT ≥ 3), surgical margin-positive or lymph node-positive cases were investigated. In terms of clinical progression, the TNC-low group in the total number of cases and among the high stage (pT ≥ 3) cases showed a worse prognosis in terms of local recurrence/metastasis. This finding is in contrast to the study of Ni et al., showing that high levels of TNC are significantly linked to lymph node metastasis and the clinical stage [40] but in agreement with another study that reported a weak TNC expression in high-grade PCa [39]. No low-risk cases or metastasis tissues were used in our study, and we focused on TNC-producing cells, not the overall TNC expression in the matrix. Therefore we can only conclude that the TNC is indeed expressed in intermediate- and high-risk primary PCa as assessed at the preoperative diagnosis based on the D´Amico criteria [17] and that a high number of TNC-positive cells is inversely correlated with clinical progression. 

More evidence points to the direction that the TNC might be degraded upon local recurrence in lung cancer [41,42], while high TNC is found in lymph and bone metastases sites [38] or even in certain types of bone metastasis [43]. In the TMA of PCa bone metastasis, San Martin et al. demonstrated a high TNC expression in trabeculae endosteum, the site of osteoblastic metastasis, and yet, a low TNC expression in the adjacent bone marrow sites [43]. Osteoblastic PCa cell lines proliferate rapidly in vitro and adhere to TNC protein, while osteolytic PC3 or lymph node-derived PCa lines do not show this phenotype, suggesting an association of TNC with osteoblastic but not osteolytic metastases. One of the ligands of TNC highly upregulated in VCap cells was α9 integrin, which binds directly TNC and a modulate expression of collagen [43], providing evidence for TNC-integrins in human PCa. Our RNA-Seq data indicate, also, the expression of α9 integrin, along with α6 and α2, and based on the proteomic human–mouse separation, we found integrin α2 to be the only one human-specific and, thus, tumor-specific for the PDXs used in this study. Although the molecular mechanism among TNC-ITGA2 should be further characterized, evidence on the correlation among α2 and α6 expressions in primary PCa and bone metastasis occurrence has been previously reported [44]. 

The reactivation of TNC expression is relevant for reactive stroma regulation, while TNC downregulation might be relevant for recurrence or metastasis initiation, which remains to be further investigated. Indeed, TNC is known to have pleiotropic functions in different cellular contexts, with both autocrine TNC expression in tumor cells and paracrine TNC from stroma in different stages of metastasis [45]; however, the cellular source of TNC in primary PCa was not addressed in our study. Our data demonstrate that androgens regulate stromal TNC expression, evident by the reduced TNC expression upon castration (even in the castration-resistant LAPC9) and immediate increased expression upon androgen replacement; thus, the TNC expression should be further evaluated in CRPC samples. Genomic amplification in the TNC gene associated with highly aggressive neuroendocrine PCa occurrence [46]. In a multi-omics approach study, the TNC protein was one of the panels of four markers detected in preoperative serum samples and, collectively, predict the biochemical relapse events with high accuracy [47]. 

In summary, we identified the stroma signature of bone metastatic PDXs, and by analyzing androgen-dependent versus androgen-independent tumors, we could demonstrate that the tumor-specific stroma gene expression changes. We could show that there are AR-regulated stromal genes modulated upon castration, even in the androgen-independent, for tumor growth, like the LAPC9 model. The osteoblastic bone metastasis stromal seven-gene signature was induced in the mouse-derived stroma compartment of BM18 and LAPC9, indicating conserved tumor mechanisms that can induce the transcriptomic “transformation” of mouse-infiltrating stroma (even in subcutaneous sites) to bone microenvironment-like stroma. The prognostic value of stroma signatures has been also demonstrated by another study utilizing PDXs associated with the metastasis prognosis from different lesions from a single PCa case and demonstrated the strong predictability of 93-gene stroma signatures to metastasis phenotypes in different clinical cohorts [21]. We identified androgen-dependent Tenascin C expression in the stroma of PDX models, which is downregulated in the conditions mimicking an aggressive disease (upon castration), similarly to the high clinical progression probability of a low TNC group in the primary PCa TMA. The higher stromal *Tnc* mRNA levels in the aggressive LAPC9 compared to BM18 may suggest that it would be relevant to examine the TNC mRNA and protein expressions in human bone metastasis or ideally matched primary metastasis cases in order to understand the kinetics of TNC in terms of disease progression. Given that TNC expression was found elevated from 0% in benign prostatic hyperplasia (BPH) stroma to 47% in tumor-associated stroma [29], its detection in circulation [47] and its immunomodulatory role [48] indicate TNC as a promising drug target and disease-determining factor. The TNC clinical progression predictive value performs best in an earlier stage, low-risk PCa, while our data show that, in high-risk PCa, a low number of TNC-producing cells were associated with poor prognosis, possibly due to changes in tissue remodeling and, thus, variable TNC levels. 

These findings were corroborated by the external clinical cohorts of patients [22,23,49,50] (Grasso et al., Lapointe et al., Taylor et al. and Varambally et al.) showing that TNC levels are downregulated during the disease progression from primary to metastasis. Based on differential expression analysis, we identified clusters of stroma signatures based on androgen-(in)dependent responses (C1-C4). TNC is a component of the C1, C3 and C4 signatures. In silico validation of the identified prostate cancer-specific stroma expression signatures on additional clinical cohorts showed the potential for patient stratification. A common feature of the majority of the four clusters of gene lists tested indicated a low stroma signature score in the advanced disease stage and a correlation with disease progression (metastasis). This was the case also for previously published stroma signatures [7,21] (Tyekucheva et al. 2017 and Mo et al. 2017) when compared to our gene sets, perhaps due to the reduced stroma content in low-differentiated, advanced PCa stage. The signature most related to the androgen-independent stage (C4) positively correlated with the Gleason score in primary tissues from TCGA but not in the metastatic cohort of the Taylor dataset. Instead, we identified a 50-gene stroma signature (C1, derived from the most androgen-responsive stroma genes), which positively correlates with the disease progression, Gleason score and poor prognosis survival, consistently on all patient cohorts evaluated, both the primary and metastasis stages. 

The regime that a metastatic, stroma-specific molecular signature may be detectable in the PCa site either prior to or during metastasis will most likely require not a single marker approach but a combination of biochemical and histological markers, taking into consideration dual tumor–stroma interactions in order to provide prognostic tools for improved patient stratification after the initial PCa diagnosis and preventive surveillance for metastasis risk. 

## 4. Materials and Methods

### 4.1. Tumor Sample Preparation and Xenograft Surgery Procedure

LAPC9 and BM18 xenografts were maintained subcutaneously in 6-week-old CB17 SCID male mice under anesthesia (Domitor^®^ 0.5 mg/kg, Dormicum 5 mg/kg and Fentanyl 0.05 mg/kg). All animal experiments were approved by the Ethical Committee of Canton Bern (animal licenses BE55/16 and BE12/17). Castration was achieved by bilateral orchiectomy. For androgen replacement, testosterone propionate dissolved in castor oil (86541-5G, Sigma-Aldrich, Buchs, Switzerland) was administered by single subcutaneous injection (2 mg per dosage, 25-G needle). 

### 4.2. RNA Isolation from Tissue Samples

Tissue RNA was extracted using the standard protocol of Qiazol (79306, Qiagen AG, Hombrechtikon, Switzerland) tissue lysis by TissueLyser (2 min, 20 Hz). Quality of RNA was assessed by Bioanalyzer 2100 (Agilent Technologies, Basel, Switzerland). RNA from formalin-fixed-paraffin embedded (FFPE) material was extracted using the Maxwell^®^ 16 LEV RNA FFPE Purification Kit (AS1260, Promega AG, Dübendorf, Switzerland). 

### 4.3. RNA Sequencing

RNA extracted from BM18, and LAPC9 whole PDX tumor extracts (300 ng) were subjected to RNA sequencing. Specimens were prepared for RNA sequencing using Tru-Seq RNA Library Preparation Kit v2 or riboZero, as previously described [51]. RNA integrity was verified using the Bioanalyzer 2100 (Agilent Technologies, Basel, Switzerland). Complementary cDNA was synthesized from total RNA using Superscript III reverse transcriptase (18080093, Thermo Fisher Scientific, Basel, Switzerland). Sequencing was then performed on GAII, Hi-Seq 2000 or Hi-Seq 2500. The sample preparation was performed according to the protocol “NEBNext Ultra II Directional RNA Library Prep Kit (NEB #E7760S/L, Illumina GmbH, Zürich, Switzerland). Briefly, mRNA was isolated from total RNA using the oligo-dT magnetic beads. After fragmentation of the mRNA, a cDNA synthesis was performed. This was used for ligation with the sequencing adapters and PCR amplification of the resulting product. The quality and yield after sample preparation was measured with the Fragment Analyzer. The size of the resulting products was consistent with the expected size distribution (a broad peak between 300–500 bp). Clustering and DNA sequencing using the NovaSeq6000 was performed according to manufacturer’s protocols. A concentration of 1.1 nM of DNA was used. Image analysis, base calling and quality check was performed with the Illumina (Illumina GmbH, Zürich, Switzerland) data analysis pipeline RTA3.4.4 and Bcl2fastq v2.20. 

Sequence reads were aligned using STAR two-pass to the human reference genome GRCh37 [52] and mouse reference genome GRCm38. Gene counts were quantified using the “GeneCounts” option. Per-gene counts-per-million (CPM) were computed and log_2_-transformed, adding a pseudo-count of 1 to avoid transforming 0. Genes with log_2_ CPM <1 in more than three samples were removed. Differential expression analysis was performed using the edgeR package [53]. Normalization was performed using the “TMM” (weighted trimmed mean) method, and differential expression was assessed using the quasi-likelihood F test. Genes with false discovery rate FDR <0.05 and >2-fold were considered significantly differentially expressed. RNA-Seq Expectation Maximization (RSEM) was used to obtain TPM (transcripts per million) counts. 

Pathway analysis (over-representation analysis) was performed using clusterProfiler R package [54] for Gene Ontology biological processes and KEGG. For Venn Euler diagram analysis, expressed genes were identified using the zFPKM transformation [55]. For the comparison between the states of the BM18 and LAPC9 models, genes were considered expressed if a gene had zFPKM values > −3 [55] in all samples.

### 4.4. Signature Validation on TCGA and Other Publically Available Datasets

TCGA gene expression, Gleason scores and outcome data were obtained from the PanCanAtlas publications supplemental data site (https://gdc.cancer.gov/about-data/publications/pancanatlas) [56,57]. For the Gene Set Variation Analysis (GSVA) analysis, RSEM expected counts in the upper quartile normalized to 1000 (i.e., the same normalization as TCGA) were used for BM18/LAPC9 gene expression. Mouse genes in gene signature lists were mapped to human homologs using the biomaRt R package (Table S5), using the “mmusculus_gene_ensembl” dataset and selecting only homologs with hsapiens_homolog_orthology_confidence = 1. Signature scores were calculated using the GSVA R package using the GSVA method [58].

Validation of the C1-C4 stroma signatures on publicly availably cohorts was performed using the Taylor (GSE21034) and the Grasso (GSE35988) datasets. Gene expression and sample information, including Gleason scores, were obtained via the GEOquery Bioconductor package. Mouse genes in the C1-C4 gene signature lists were mapped to human homologs using the biomaRt R package, using the “mmusculus_gene_ensembl” dataset and selecting only homologs with hsapiens_homolog_orthology_confidence = 1. Other gene sets are either human genes or include info on human homologs. Signature scores were calculated using the GSVA R package using the GSVA method [58].

### 4.5. Tissue Dissociation and MACS

Tumor tissue was collected in a basis medium (advanced Dulbecco Modified Eagle Medium F12 serum-free medium (12634010, Thermo Fisher Scientific, Basel, Switzerland) containing 10-mM Hepes (15630080, Thermo Fisher Scientific, Basel, Switzerland), 2-mM GlutaMAX supplement (35050061, Thermo Fisher Scientific, Basel, Switzerland) and 100 μg/mL Primocin (ant-pm-1, InVivoGen, LabForce AG, Muttenz, Switzerland). After mechanical disruption, the tissue was washed in the basis medium (220 relative centrifugal force (rcf), 5 min) and incubated in the enzyme mix for tissue dissociation (collagenase type II enzyme mix (17101-015, Gibco, Thermo Fisher Scientific, Basel, Switzerland) 5 mg/mL dissolved in the basis medium and DNase: 15 μg/mL (10104159001, Sigma-Aldrich, Buchs, Switzerland) and 10-μM Y-27632-HCl rock inhibitor (S1049, Selleckchem, Zürich, Switzerland). Enzyme mix volume was adjusted so that the tissue volume did not exceed 1/10 of the total volume, and tissue was incubated at 37 °C for 1 to 2 h, with mixing every 20 min. After the digestion of large pieces was complete, the suspension was passed through a 100-μm cell strainer (21008-950, Falcon^®^, VWR International GmbH, Dietikon, Switzerland) attached to a 50-mL Falcon tube, then using a rubber syringe to crash tissue against the strainer and wash in 5-mL basic medium (220 rcf, 5 min). Cell pellet was incubated in 5-mL precooled red blood cell lysis buffer (150-mM NH_4_Cl, 10-mM KHCO_3_ and 0.1-mM EDTA), incubated for 10 min and washed in equal volume of basis medium, followed by centrifugation (220 rcf, 5 min). Pellet was resuspended in 2–5 mL accutase™ (StemCell Technologies, 07920), depending on the sample amount; biopsies versus tissue and incubated for 10 min at room temperature. The cell suspension was passed through a 40-μm pore size strainer (21008-949, Falcon^®^, International GmbH, Dietikon, Switzerland), and the strainer was washed by adding 2 mL of accutase on the strainer. Single-cell suspension was counted to determine the seeding density and washed in 5 mL of basis medium and spun down 220 rcf, 5 min. Magnetic cell sorting was performed to separate purified human versus mouse cell fractions using the Mouse Cell Depletion Kit (130-104-694, Miltenyi Biotek, Solothurn, Switzerland). For the proteomic experiments, cell fractions from tumor tissues (*n* = 3 to 4 per condition) were pooled together in order to suffice for 10^6^ cells, representing one technical replicate per sample. 

### 4.6. Proteomics

#### 4.6.1. Sample Preparation

Approx. 10^6^ cell pellets (*n* = 1 technical replicate per condition deriving from *n* = 3 to 4 biological replicate samples) were resuspended in 50 µL PBS following the addition of 50 µL 1% SDS in 100-mM Hepes/NaOH, pH 8.5 supplemented with protease inhibitor cocktail EDTA-free (11836170001, Sigma-Aldrich, Buchs, Switzerland). Samples were heated to 95 °C for 5 min, transferred on ice, and benzonase (71206-3, Merck AG, Zug, Switzerland) was added to degrade DNA at 37 °C for 30 min. Samples were reduced by the addition of 2 µL of a 200-mM DTT solution in 200-mM Hepes/NaOH, pH 8.5 and, subsequently, alkylated by the addition of 4 µL of a 400-mM chloroacetamide (CAA, #C0267, Sigma-Aldrich, Buchs, Switzerland) solution in 200 mM Hepes/NaOH, pH 8.5. Samples were incubated at 56 °C for 30 min. Access CAA was quenched by the addition of 4 µL of a 200-mM DTT solution in 200 mM Hepes/NaOH, pH 8.5. Lysate were subjected to an in-solution tryptic digest using the single-pot solid phase-enhanced sample preparation (SP3) protocol [59,60]. To this end, 20 µL of Sera-Mag Beads (#4515-2105-050250 and 6515-2105-050250, Thermo Fisher Scientific, Basel, Switzerland) were mixed, washed with H_2_O and resuspended in 100 µL H_2_O. Two microliters of freshly prepared bead mix and 5 µL of an aqueous 10% formic acid were added to 40 µL of lysates to achieve an acidic pH. Forty-seven microliters of acetonitrile were added, and samples were incubated for 8 min at room temperature. Beads were captured on a magnetic rack and washed three times with 70% ethanol and once with acetonitrile. Sequencing grade-modified trypsin (0.8 µg; V5111, Promega AG, Dübendorf, Switzerland) in 10 µL 50 mM Hepes/NaOH, pH 8.5 were added. Samples were digested overnight at 37 °C. Beads were captured and the supernatant transferred and dried down. Peptides were reconstituted in 10 µL of H_2_O and reacted with 80 µg of TMT10plex (#90111, Thermo Fisher Scientific, Basel, Switzerland) [61] label reagent dissolved in 4 µL of acetonitrile for 1 h at room temperature. Excess TMT reagent was quenched by the addition of 4 µL of an aqueous solution of 5% hydroxylamine (438227, Sigma-Aldrich, Buchs, Switzerland). Mixed peptides were subjected to a reverse-phase clean-up step (OASIS HLB 96-well µElution Plate, 186001828BA, Waters Corporation, Milford, MA, USA) and analyzed by LC-MS/MS on a Q Exactive Plus (Thermo Fisher Scientific, Basel, Switzerland), as previously described [62].

#### 4.6.2. Mass Spectrometric Analysis

Briefly, peptides were separated using an UltiMate 3000 RSLC (Thermo Scientific, Basel, Switzerland) equipped with a trapping cartridge (Precolumn; C18 PepMap 100, 5 Lm, 300 Lm i.d. × 5 mm, 100 A°) and an analytical column (Waters nanoEase HSS C18 T3, 75 Lm × 25 cm, 1.8 Lm, 100 A°). Solvent A: aqueous 0.1% formic acid and Solvent B: 0.1% formic acid in acetonitrile (all solvents were of LC-MS grade). Peptides were loaded on the trapping cartridge using solvent A for 3 min with a flow of 30 µL/min. Peptides were separated on the analytical column with a constant flow of 0.3 µL/min applying a 2 h gradient of 2–28% of solvent B in A, followed by an increase to 40% B. Peptides were directly analyzed in positive ion mode, applied with a spray voltage of 2.3 kV and a capillary temperature of 320°C using a Nanospray-Flex ion source and a Pico-Tip Emitter 360 Lm OD × 20 Lm ID;, 10 Lm tip (New Objective, Littleton, MA, USA). MS spectra with a mass range of 375–1.200 m/z were acquired in profile mode using a resolution of 70,000 (maximum fill time of 250 ms or a maximum of 3 × 10^6^ ions (automatic gain control, AGC)). Fragmentation was triggered for the top 10 peaks with 2–4 charges on the MS scan (data-dependent acquisition), with a 30 s dynamic exclusion window (normalized collision energy was 32). Precursors were isolated with a 0.7 m/z window and MS/MS spectra were acquired in profile mode with a resolution of 35,000 (maximum fill time of 120 ms or an AGC target of 2 × 10^5^ ions).

#### 4.6.3. Raw MS Data Analysis

Acquired data were analyzed using IsobarQuant [63] and Mascot V2.4 (Matrix Science, Chicago, IL, USA) using either a reverse-UniProt FASTA Mus musculus (UP000000589) or Homo sapiens (UP000005640) database. Moreover, a combined database thereof was generated and used for the analysis. These databases also included common contaminants. The following modifications were taken into account: Carbamidomethyl (C, fixed), TMT10plex (K, fixed), Acetyl (N-term, variable), Oxidation (M, variable) and TMT10plex (N-term, variable). The mass error tolerance for full-scan MS spectra was set to 10 ppm and for MS/MS spectra to 0.02 Da. A maximum of 2 missed cleavages were allowed. A minimum of 2 unique peptides with a peptide length of at least seven amino acids and a false discovery rate below 0.01 were required on the peptide and protein levels [64].

#### 4.6.4. MS Data Analysis

The raw output files of IsobarQuant (protein.txt files) were processed using the R programming language (ISBN 3-900051-07-0). As a quality filter, only proteins were allowed that you were quantified with at least two unique peptides. Human and mouse samples were searched against a combined human and mouse database and annotated as unique for human or mouse or mixed. Raw signal-sums (signal_sum columns) were normalized using vsn (variance stabilization normalization) [65]. In order to try to annotate each observed ratio with a *p*-value, each ratio distribution was analyzed with the locfdr function of the locfdr package [66] to extract the average and the standard deviation (using the maximum likelihood estimation). Then, the ratio distribution was transformed into a z-distribution by normalizing it by its standard deviation and mean. This z-distribution was analyzed with the fdrtool function of the fdrtool package [67] in order to extract *p*-values and false discovery rates (fdr, *q*-values). 

### 4.7. Tissue Microarray

Tissue microarray core annotations and quantification of positive staining were performed by QuPath software version v0.2.0-m8 [68] using the TMA map function. Kaplan–Meier curves to calculate the association between TNC-positive cells and disease progression were calculated using the “survfit” function and the global Log-Rank test using the Survival R package [69,70]. To estimate the survival, we used the function “surv_cutpoint”, which employs maximally selected rank statistics (maxstat) to determine the optimal cut-point for continuous variables [18]. For pairwise comparison, the *p*-value was estimated by the Log-Rank test and adjusted with the Benjamini–Hochberg (BH) method. If no information on patient outcome was available, information at the last follow-up was used for all parameters. Clinical progression was defined as metastasis or local recurrence. Disease progression was defined by combining any form of recurrence (PSA and clinical progression). Data representation and graphical plots were generated using the ggplot2 R package [71]. Data analyses were done using RStudio version 1.1.463 [72] and R version 3.5.3 [73].

### 4.8. Immunohistochemistry

FFPE sections (4 μm) were deparaffinized and used for heat-mediated antigen retrieval (citrate buffer, pH 6, Vector Labs). Sections were blocked for 10 min in 3% H_2_O_2_, followed by 30 min, RT incubation in 1% BSA in PBS–0.1%Tween 20. The following primary antibodies were used (Table 3): 

Secondary anti-rabbit antibody Envision HRP (DAKO, Agilent Technologies, Basel, Switzerland) for 30 min or anti-rat HRP (Thermo Scientific, Basel, Switzerland). Signal detection with AEC substrate (DAKO, Agilent Technologies, Basel, Switzerland). Sections were counterstained with Hematoxylin and mounted with Aquatex.

### 4.9. Immunofluorescence

After deparaffinization, heat-mediated antigen retrieval (citrate buffer, pH 6, Vector Labs) was performed. Sections were blocked in 1% BSA in PBS–0.1% Tween 20 for 30 min, RT incubation. The primary antibodies used (Table 4), were incubated overnight in blocking solution at 4 °C:

Secondary anti-rabbit/mouse/goat/rat antibodies coupled to Alexa Fluor^®^-488, 555 or 647 fluorochrome conjugates (Invitrogen, Thermo Scientific, Basel, Switzerland) were incubated for 90 min at 1:250 dilution in PBS. Sections were counterstained with DAPI solution (Thermo Scientific, Basel, Switzerland, final concentration 1 μg/mL in PBS, 10 min), washed and mounted with prolonged diamond antifade reagent (Invitrogen, Thermo Scientific, Basel, Switzerland). 

## 5. Conclusions

In this proof-of-concept study, the molecular profile of the stroma in prostate cancer was shown to be responsive to androgen deprivation even in advanced, androgen-independent bone metastasis prostate cancer. We identified a stroma-specific gene expression signature that correlates with the Gleason score and metastatic disease progression of prostate cancer. Given the inevitable drug resistance to androgen deprivation therapies, stroma biomarker identification associated with resistance acquisition may complement standard histopathology and genomic evaluations for improved stratification of patients at high risk. 

## Figures and Tables

**Table 1:** Clinical parameters of the EMPaCT TMA patient cases.

Descriptive Statistics	Age at Surgery	PSA at Surgery	PSA Progression Time (Months)	Clinical Progression Time (Months)
**Min**	43	20	1	1
**1st quartile**	62	25.33	29.5	40.5
**Median quartile**	67	36.99	63.5	75.5
**Mean quartile**	66.18	50.56	63.47	70.89
**3rd quartile**	71	61.9	90	95.75
**Max quartile**	81	597	151	153

**Table 2:** Pathological staging, PSA and Clinical Progression of the EMPaCT TMA patient cases.

PSA Progression	Clinical Progression	Pathological Staging (No. of Patient Cases)				
		2a	2b	3a	3b	4
**no**	no	6	15	37	63	26
	yes	0	0	0	1	0
**yes**	no	0	5	7	11	7
	yes	1	7	9	9	6

**Table 3:** Primary antibodies used for Immunohistochemistry

Dilution	Antibody	Company	Catalog No.
1 to 500	Ki67	Gene Tex	GTX16667
1 to 100	Tnc	R&D	MAB2138

**Table 4:** Primary antibodies used for Immunofluorescence

Dilution	Antibody	Company	Catalog No.
1 to 500	αSMA	Sigma	A2547
1 to 500	ITGA2	Abcam	ab181548
1 to 500	Collagen type I	Southern Biotech	1310-01
1 to 50	Tnc	R&D	MAB2138

## Data Availability

The sequencing data have been submitted to the European Genome-Phenome Archive under the accession number EGAS00001004770.

## Supplementary Materials

The following are available online at https://www.mdpi.com/2072-6694/12/12/3786/s1: Figure S1. Related to Figure 2. Human transcriptomic profile of BM18 and LAPC9 tumors. Figure S2. Related to Figure 2. Human and mouse ratios of RNA-Seq transcript levels in intact and castrated settings. Figure S3. Related to Figure 5. Pathways enriched in the stroma of the CRPC LAPC9 compared to the BM18. Figure S4. Related to Figure 7. PSA progression in cases with positive surgical margins or lymph nodes status. Figure S5. Related to Figure 9. Correlations and prognostic performances of the C1, C2, C3 and C4 signatures compared to the previously identified stroma signatures. Table S1. Venn Euler diagrams. Table S2: Lists of enriched GO and KEGG pathways (attached as an Excel file). Table S3. Statistical test TNC expression in patient groups of different pT Stage classification in the high risk PCa TMA. Table S4. Stroma signature gene lists. Table S5. Human gene lists of Ob-BMST and C1-C4 signatures.
