# Supplementary material for: Stroma Transcriptomic and Proteomic Profile of Prostate Cancer Metastasis Xenograft Models Reveals Prognostic Value of Stroma Signatures
Source: Cancers (Basel). 2020 Dec 15;12(12):3786. doi: 10.3390/cancers12123786 (PMC7768471; doi:10.3390/cancers12123786)
Supplement: Supplementary file 1 [file cancers-12-03786-s001.pdf]

Supplementary Materials: Stroma Transcriptomic and Proteomic Profile of Prostate Cancer Metastasis Xenograft Models Reveals Prognostic Value of Stroma Signatures

Sofia Karkampouna, Maria R. De Filippo, Charlotte K.Y. Ng, Irena Klima, Eugenio Zoni, Martin Spahn, Frank Stein, Per Haberkant, George N.Thalmann and Marianna Kruithof de Julio

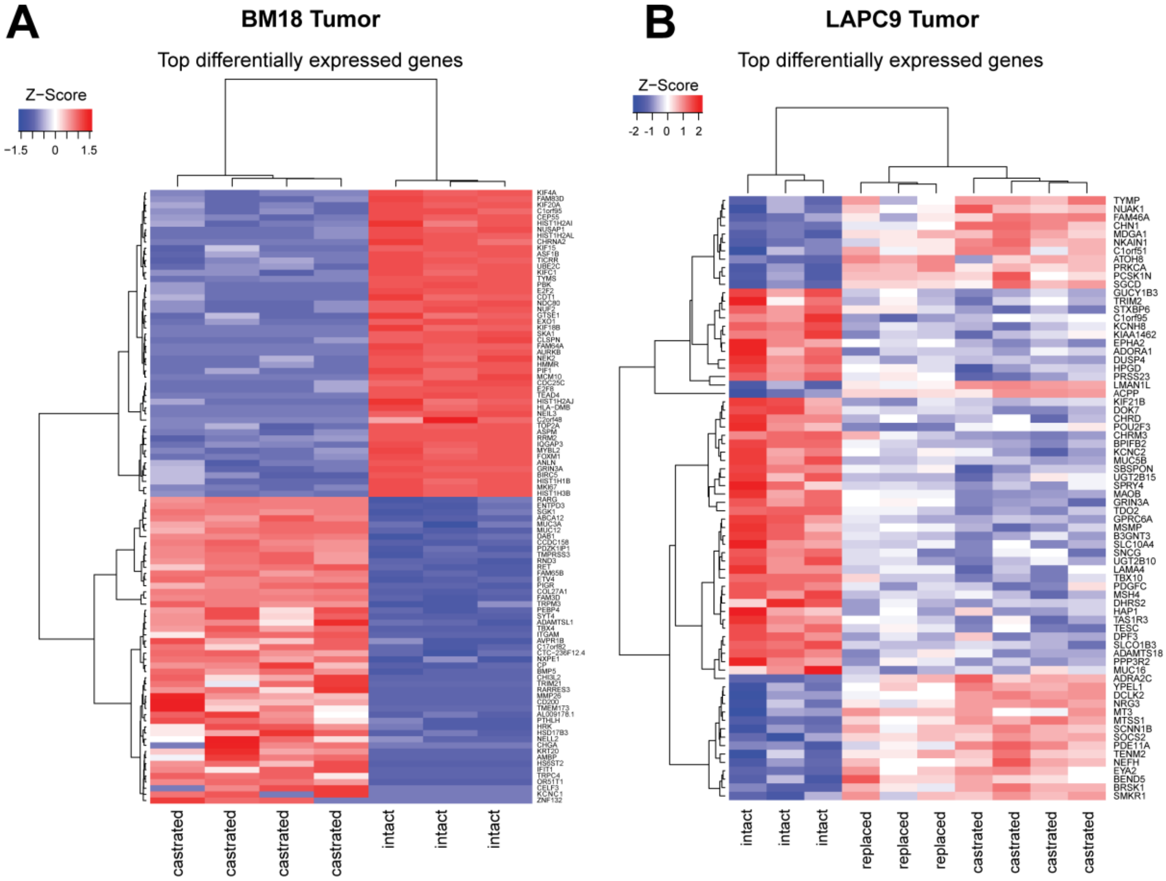

Figure S1. Related to Figure 2. Human transcriptomic profile of BM18 and LAPC9 tumors. (**A**) Heatmap represents the differential expression analysis of most variable genes from the human transcriptome of BM18 castrated compared to BM18 intact tumors. (**B**) Heatmap represents the differential expression analysis of most variable genes from the human transcriptome of LAPC9 castrated (with and without androgen replacement) compared to LAPC9 intact tumors. Genes modulated under androgen deprivation conditions by up/downregulation compared to intact tumors are indicated in red or blue color, respectively.

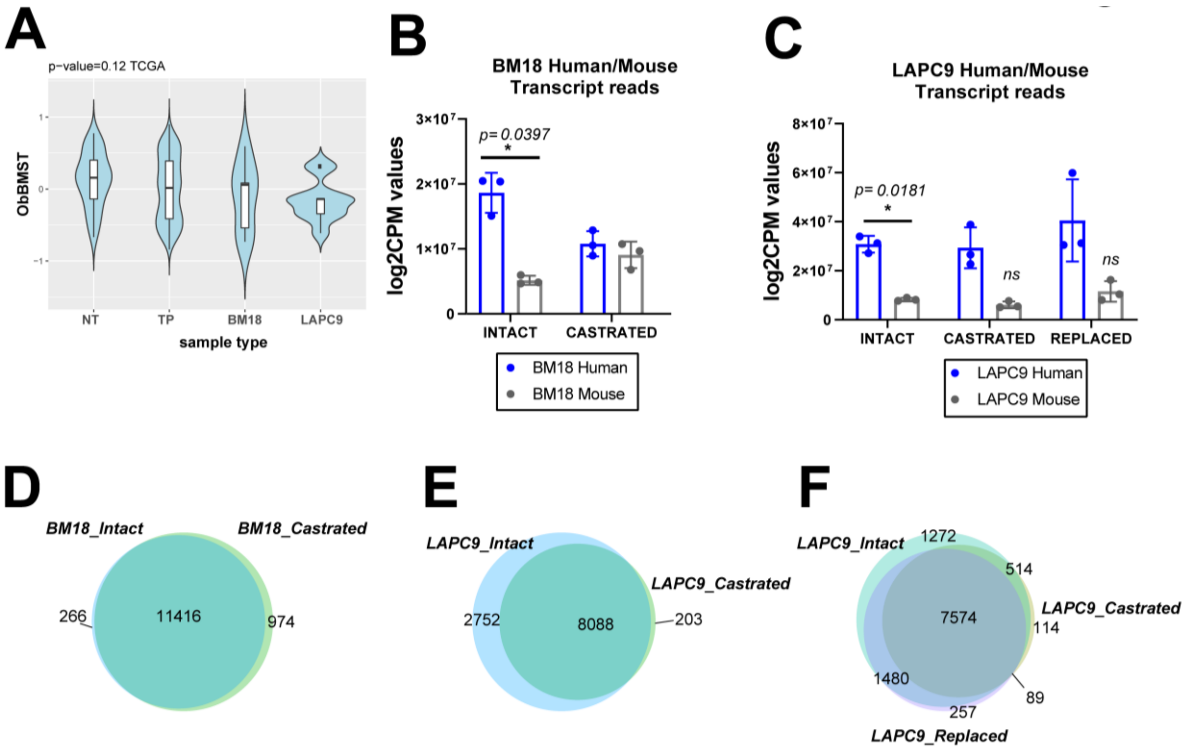

Figure S2. Related to Figure 2. Human and mouse ratios of RNA-Seq transcript levels in intact and castrated settings. (**A**) OB-BMST stroma signature gene expression in the BM18, LAPC9 and TCGA cohorts (normal, NT and primary tumor, TP). Violin plot showing GSVA signature scores of the gene sets, stratified by sample types (NT = nontumor and TP = primary tumor). Box-and-whisker plots illustrating the median (midline) and interquartile range (box), with the whiskers extending to, at most, 1.5 IQR from the box. Outliers beyond the range of the whiskers are illustrated as dots. *P*-values for first figure computed by the Kruskal-Wallis test. (**B**) Human and mouse ratios of RNA transcript levels (log_2CPM values) of BM18 (intact and castrated) and (**C**) of LAPC9 (intact, castrated and replaced). (**D**–**F**) Venn Euler diagrams showing the number of expressed mouse genes in (**D**) BM18 (intact and castrated), (**E**) LAPC9 (intact and castrated) and (**F**) LAPC9 (intact, castrated and replaced). Full lists of overlapping genes in Table S1.

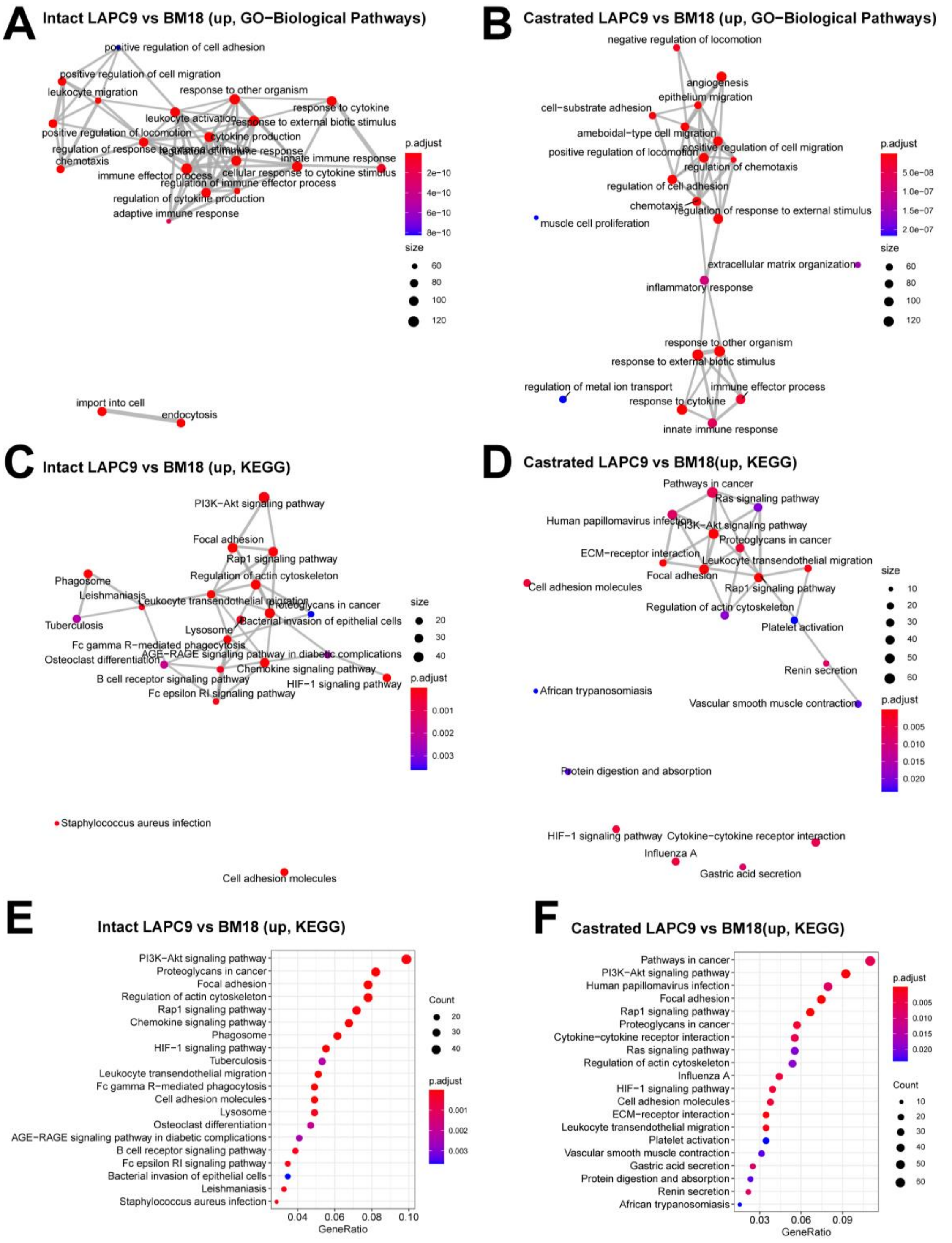

Figure S3. Related to Figure 5. Pathways enriched in the stroma of the CRPC LAPC9 compared to BM18. (**A–D**) Enrichment maps of the top 20 enriched pathways for the respective groups and pathway sets (GO terms biological pathways and KEGG). (**E**,**F**) Dot plots of the top 20 enriched pathways for the respective groups in the KEGG pathway set. GeneRatio corresponds to the fraction of differentially expressed genes belonging to a given pathway. Full lists of enriched pathways are in Table S2.

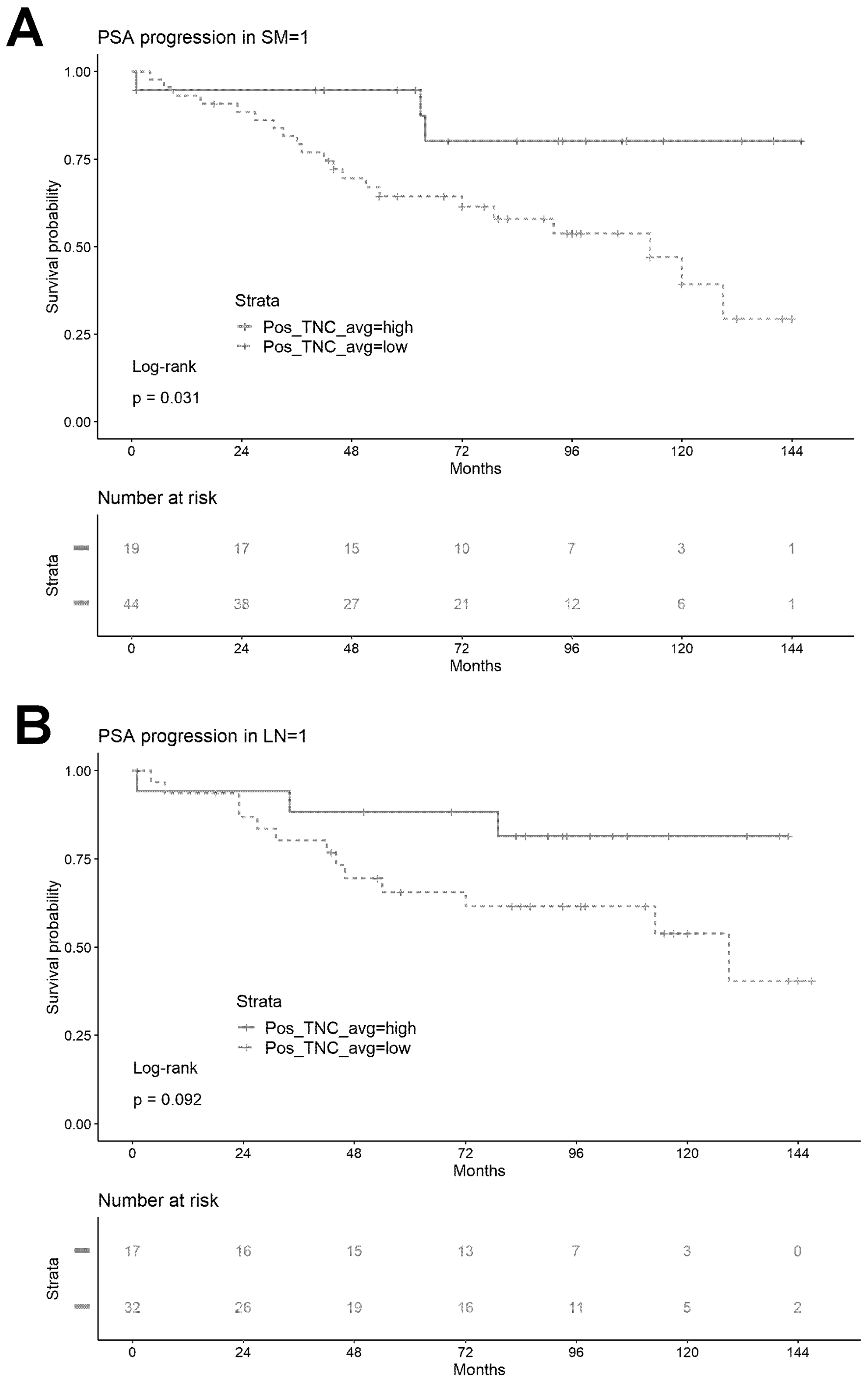

Figure S4. Related to Figure 7. PSA progression in cases with positive surgical margins or lymph node status. (**A**) PSA progression probability of patient cases with a positive surgical margin presence (SM = 1), according to TNC-high and TNC-low expressions (p = 0.031 and *<0.05). (**B**) PSA progression probability of patient cases with positive lymph nodes (LN = 1) status according to TNC-high and TNC-low expressions (p = 0.092 and ns (not significant)).

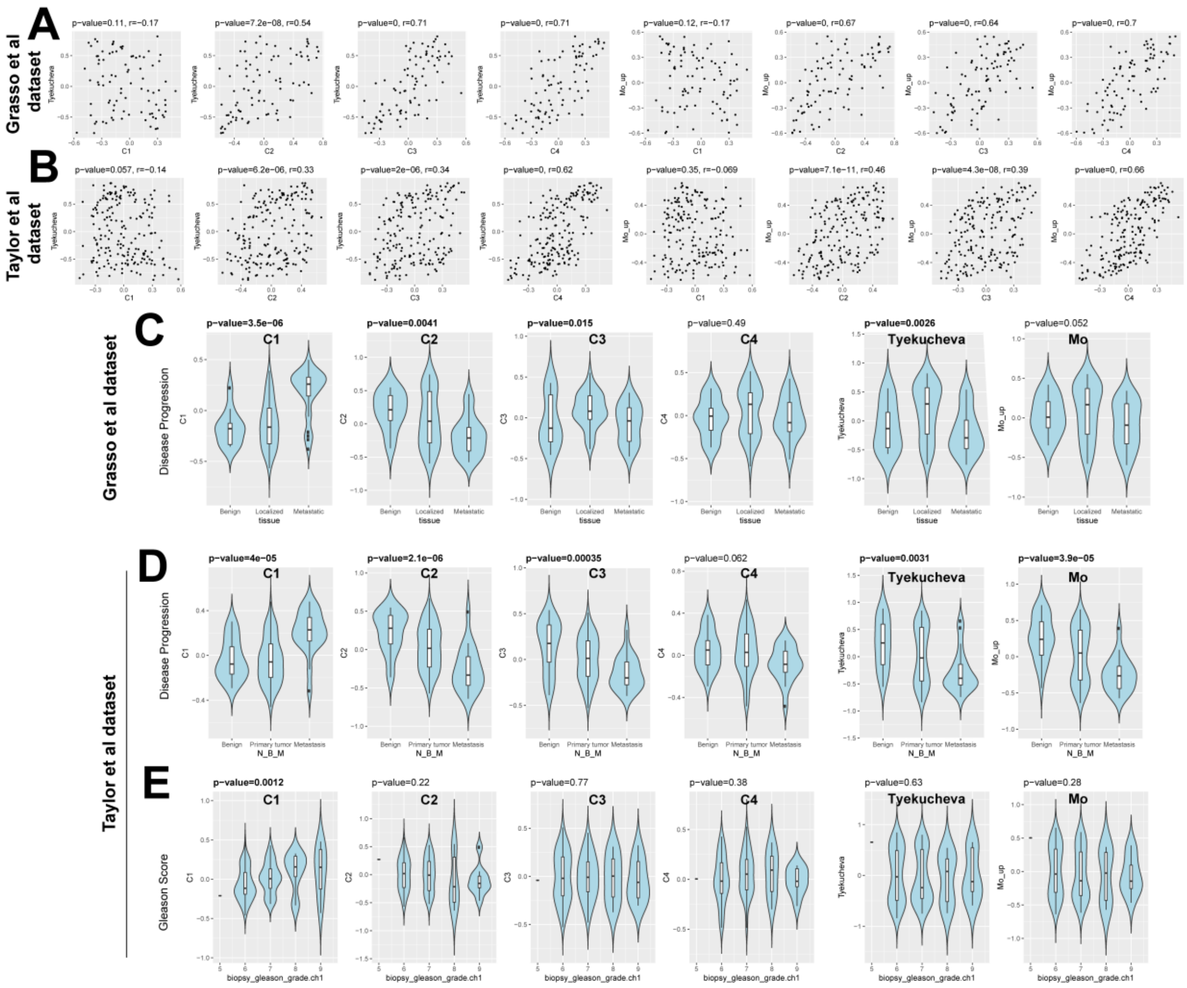

Figure S5. Related to Figure 9. Correlations and prognostic performance of the C1, C2, C3 and C4 signatures compared to previously identified stroma signatures. (**A**) Scatterplot illustrating the correlation between C1-4 and the other 2 signatures identified by previous studies [1,2] (Tyekucheva et al. and Mo et al.) across two different patient cohort datasets: the Grasso dataset (GSE35988) and (**B**) Taylor dataset (GSE21034). Correlation assessed by Spearman correlation tests. (**C**) Violin plots showing GSVA signature scores of the gene sets, stratified by benign, primary and metastases groups from the Grasso et al. dataset (Kruskal-Wallis test). (**D**) Violin plots showing GSVA signature scores of the gene sets, stratified by benign, primary and metastases groups (Kruskal-Wallis test), and (**E**) intermediate-to-high Gleason scores (GS6-10) from the Taylor et al. dataset [3] (Spearman correlation test).

Supplementary Table S1. Venn Euler diagrams. Data used for the generated Venn diagrams, where TRUE indicates that the gene is expressed (zFPKM > −3) in all samples of the given group (attached as an Excel file).

Supplementary Table S2: Lists of enriched GO and KEGG pathways (attached as an Excel file).

Supplementary Table S3. Statistical test of TENASCIN C expression in the patient groups of different pT Stage classifications in the high-risk prostate cancer (PCa) TMA. Statistical test outcomes related to the TMA data in Figure 7. Shapiro statistical test for pathologic tumor stage (pT) Stage 3a, 3b, ($p <$ 0.001 ****) and group 4 ($p > 0.05$, nonsignificant). Data not homogeneously distributed; therefore, a nonparametric one-way ANOVA and multiple comparisons using the Wilcoxon rank sum test among the pT Stage groups (2a, 2b, 3a, 3b and 4) were performed. Adjusted *p*-values indicated $p > 0.05$, ns.

Pairwise Comparisons Using the Wilcoxon Rank Sum Test				
	2a	2b	3a	3b
2b	0.86	-	-	-
3a	1.00	0.86	-	-
3b	1.00	0.86	0.86	-
4	1.00	0.86	1.00	1.00

p-value adjustment method: BH

Supplementary Table S4. Stroma signature gene lists. Gene lists of the Ob-BMST signature. Clusters of signature lists: C1, C2, C3 and C4. Signature lists from Tyekucheva et al. (signature enriched for genes expressed in high-grade tumor-associated stroma) and Mo et al. (signature of upregulated genes distinguishing metastatic progression) (attached as an Excel file).

Supplementary Table S5. Human gene lists of Ob-BMST and C1-C4 signatures. Mouse signature lists were mapped to human homologs using the biomaRt R package (attached as an Excel file).

References

1. Tyekucheva, S.; Bowden, M.; Bango, C.; Giunchi, F.; Huang, Y.; Zhou, C.; Bondi A, Lis R, Van Hemelrijck M, Andrén O; et al. Stromal and epithelial transcriptional map of initiation progression and metastatic potential of human prostate cancer. *Nat. Commun.* **2017**, *8*, 420.
2. Mo, F.; Lin, D.; Takhar, M.; Ramnarine, V.R.; Dong, X.; Bell, R.H. Volik SV, Wang K, Xue H, Wang Y; et al. Stromal gene expression is predictive for metastatic primary prostate cancer. *Eur. Urol.* **2018**, *73*, 524–532.
3. Taylor, B.S.; Schultz, N.; Hieronymus, H.; Gopalan, A.; Xiao, Y.; Carver, B.S.; Arora VK, Kaushik P, Cerami E, Reva B; et al. Integrative genomic profiling of human prostate cancer. *Cancer Cell.* **2010**, *18*, 11–22.

© 2020 by the authors. Licensee MDPI, Basel, Switzerland. This article is an open access article distributed under the terms and conditions of the Creative Commons Attribution (CC BY) license (http://creativecommons.org/licenses/by/4.0/).
